# *In situ* synthesis of high-quantum-efficiency and stable bromide-based blue-emitting perovskite nanoplatelets†

**DOI:** 10.1039/d2na00354f

**Published:** 2022-10-13

**Authors:** Srinivasa Rao Pathipati, Muhammad Naeem Shah, Syed Akhil, Nimai Mishra

**Affiliations:** Laboratory for Semiconductor Research, Department of Physics, School of Applied Science and Humanities, Vignan's Foundation for Science, Technology, and Research (Deemed University) Vadlamudi Guntur Andhra Pradesh India 522213 srinivasphm@gmail.com drsrp_sh@vignan.ac.in; College of Electronics and Information Engineering, Shenzhen University Shenzhen Guangdong P. R. China 518000; Department of Chemistry, SRM University – AP, Andhra Pradesh Neerukonda, Guntur Andhra Pradesh 522240 India

## Abstract

We present a facile synthetic approach for the growth of two-dimensional CsPbBr_3_ nanoplatelets (NPLs) in the temperature range of 50–80 °C *via* the vacuum-assisted low-temperature (VALT) method. In this method, we utilized the solubility of the PbBr_2_ precursor at temperatures high than the reaction temperature, thus making Br available during the reaction to form NPLs with fewer defects. The high chemical availability of Br during the reaction changes the growth dynamics and formation of highly crystalline nanoplatelets. Using this method, we have synthesized NPLs with an emission wavelength range of 450 to 485 nm that have high photoluminescence quantum yields (PLQY) from 80 to 100%. The synthesized NPLs retain their initial PLQY of about 80% after one month at ambient conditions. The formation of NPLs with fewer defects and enhanced radiative recombination was further confirmed by X-ray diffraction (XRD), reduced Urbach energy, time-resolved photocurrent measurements, X-ray photoelectron spectroscopy (XPS), and Fourier-transform infrared (FTIR) spectroscopy. Additionally, we utilized the synthesized NPLs for the fabrication of down-conversion light emitting diodes (LEDs), and the electroluminescence peak was barely shifted compared to the photoluminescence peak.

## Abstract 2

We present a facile synthetic approach for the growth of two-dimensional CsPbBr_3_ nanoplatelets (NPLs) in the temperature range of 50–80 °C *via* the vacuum-assisted low-temperature (VALT) method.

## Graphical abstract

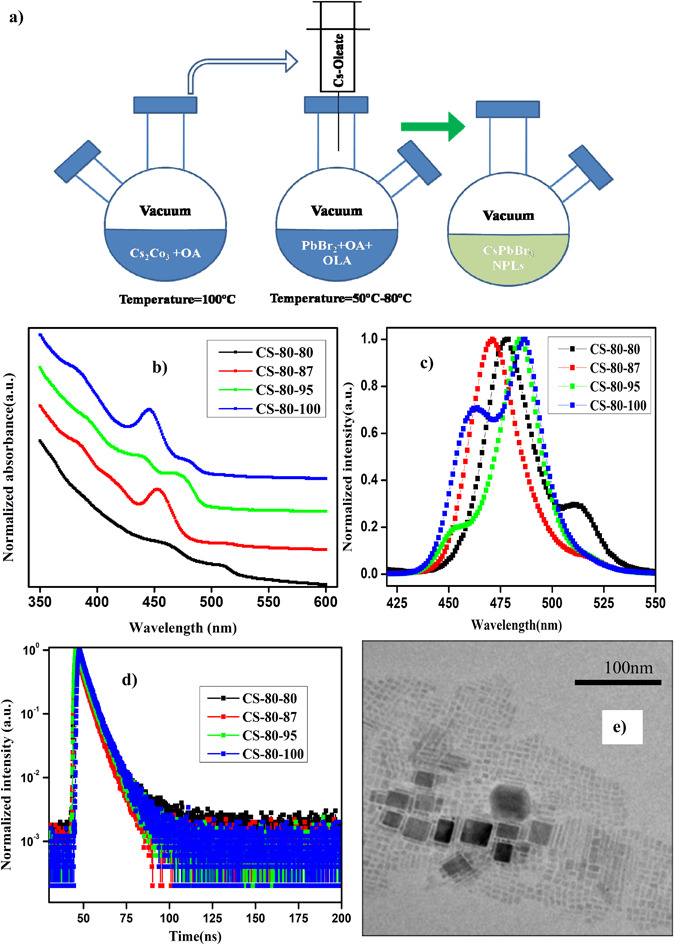

## 1. Introduction

Two-dimensional (2D) perovskite nanoplatelets (NPLs) have attracted a great deal of attention due to their superior electrical and optical properties^1–4^ and couple two fundamental areas of research, namely, 2D materials and lead halide perovskites.^5,6^ The photoluminescence quantum yield (PLQY) of blue-emission nanocrystals (NCs) is lagging compared to those of green- and red-light-emitting NCs.^7–13^ This is mainly due to two reasons: (i) the chloride-containing NCs exhibit poor PLQY in the blue emission range,^14,15^ and (ii) mixed-halide perovskites have phase segregation into large crystals, which results in shifting of the emission wavelength during the operation of the device.^16^ Another viable approach for strong blue emission is to control the bandgap of cubic-shaped CsPbBr_3_ NCs, which can emit at 530 nm.^17,18^ The bandgap of CsPbBr_3_ can be increased by controlling the dimensionality from 3D to 2D NPLs. It has been demonstrated that the thickness of NPLs can be controlled from a few monolayers to a single monolayer with atomic precision by controlling the reaction temperature,^19^ ligand concentration,^5,20^ and the ratio of the precursors.^21^ However, these NPLs have a high surface-to-volume ratio, which results in more atoms on the surface. Hence, these NPLs are more susceptible to surface defects, which results in low PLQY values when the morphology is changed from a cubic to a platelet structure.^22,23^ For instance, the PLQY drops from 90–100% to 20–30% when the morphology changes from cubic to thin NPLs.^24,25^ The low PLQY mainly results from a large number of defects on the surface and non-stoichiometric composition.^21,23^ Additionally, the morphology of the NPLs changes with time and results in ripening and aggregation, which consequently result in PL peak shift and broad emission line widths.^26^ For successful exploitation of NPL-based devices, it is necessary to develop techniques to obtain simultaneously highly emissive and stable perovskite materials.^23,27,28^ Thus, much work is needed for the synthesis of defect-free highly luminescent blue-emitting perovskite NPLs. Several strategies have been developed to obtain high PLQY, which include post-synthesis surface passivation,^21,29,30^ metal ion doping,^31^ and modified synthesis procedures.^26,30,32,33^ The first report on the synthesis of NPLs was reported by Bekenstein *et al.* using the conventional hot injection method.^19^ Later, some room-temperature synthesis approaches were developed, such as the reprecipitation method^21^ and the ligand-assisted liquid-phase exfoliation method.^34^ However, the solubility of PbBr_2_ in the room-temperature processing methods is limited, which limits the chemical availability of Br and the formation of PbBr_6_^4−^ octahedra. Additionally, the complex nucleation and growth mechanism introduces enormous amounts of surface defects, which result in trap states. These trap states will increase the recombination rate of the charge carriers and decrease the PLQY. To improve the PLQY of the NPLs through passivation of the surface defects, several strategies have been developed, such as post-synthetic trap repair with a PbBr_2_-ligand solution.^7,21^ Similarly, the addition of HBr to the precursor solvent itself during the reaction would improve the chemical availability of Br, which helps to change the growth dynamics and to form the highly crystalline NPLs.^5,26^ The lead halide perovskite structure consists of Cs ions occupying the corners and Pb and Br atoms occupying the body center and face-centered positions, respectively. The Pb and Br ions assemble into octahedra (PbBr_6_^4−^), and the Br 4p and Pb 6p orbitals mainly contribute to the upper valence and conduction band, respectively.^35,36^ The charge carrier excitation and recombination process mainly occurs within the octahedra, and hence the Br and Pb vacancies introduce trap states, which result in wide full-width half-maximum (FWHM) and low PLQY values.^36,37^ These surface defects reduce not only the PLQY, but also the stability, of the NCs during the purification process.^38^ Hence, reducing the defect density of Br is one of the most important viable approaches to improve the PLQY of the NPLs. Since the halide perovskites are ionic, strong interactions exist among the ions, which results in fast nucleation rates of less than 1 ms.^39^ The nuclei start to grow as the atoms from the surroundings become attached to them. The chemical availability and the coordination chemistry during the crystallization process have decisive significance to the final structure of the NCs.^40,41^ The ligands and coordinated solvents compete with the halide ions while forming the PbBr_6_^4−^ octahedra, which results in the formation of Br defects. Therefore, a greater amount of Br ions before the nucleation begins will result in fewer defects, and this *in situ* passivation approach might be one of the easiest ways to improve the PLQY.^26,42^ From previous reports, it is evident that the addition of excess Br during the reaction changes the growth dynamics of NCs as well as their size and shape.^17,43^ Dong *et al.* synthesized NPLs by the addition of trivalent ions and controlled the thickness and the emission in the blue region.^44^ Similarly, Xiao *et al.* reported that the addition of metal bromide changes the growth dynamics and forms the NPLs.^45^ Furthermore, Dutta *et al.* showed that the size of the NCs can be fine-tuned *via* the HBr content.^46^

In this work, we have synthesized NPLs using the vacuum-assisted low-temperature (VALT) method. This method has the advantage of a relatively low processing temperature of about 40 °C compared to the conventional hot-injection method. Additionally, the NCs were grown in a vacuum environment without any use of a Schlenk line or inert atmosphere.^47^ These synthesized NPLs can emit in the wavelength range from 450–485 nm. Here, we propose an *in situ* synthesis process to form PbBr_6_^4−^ octahedra before nucleation begins. To improve the chemical availability of Br to form PbBr_6_^4−^ octahedra during the reaction, we have utilized the solubility of PbBr_2_ at higher temperatures. Hence, we heated the PbBr_2_ precursor to a higher temperature than the reaction temperature for a sufficient time, thereby improving the solubility and increasing the chemical availability of Pb and Br in the reaction mixture. In the proposed *in situ* passivation mechanism, the PbBr_2_ precursor is heated to various temperatures higher than the reaction temperature. Consequently, blue-emitting NPLs with an emission wavelength of 450 nm are obtained with a PLQY of 80–90%. These NPLs retain their initial PLQY of about 80% when exposed to the ambient atmosphere, even after one month.

## 2. Experimental details

### 2.1 Synthesis of CsPbBr_3_ NPLs

**Fig. 1:**
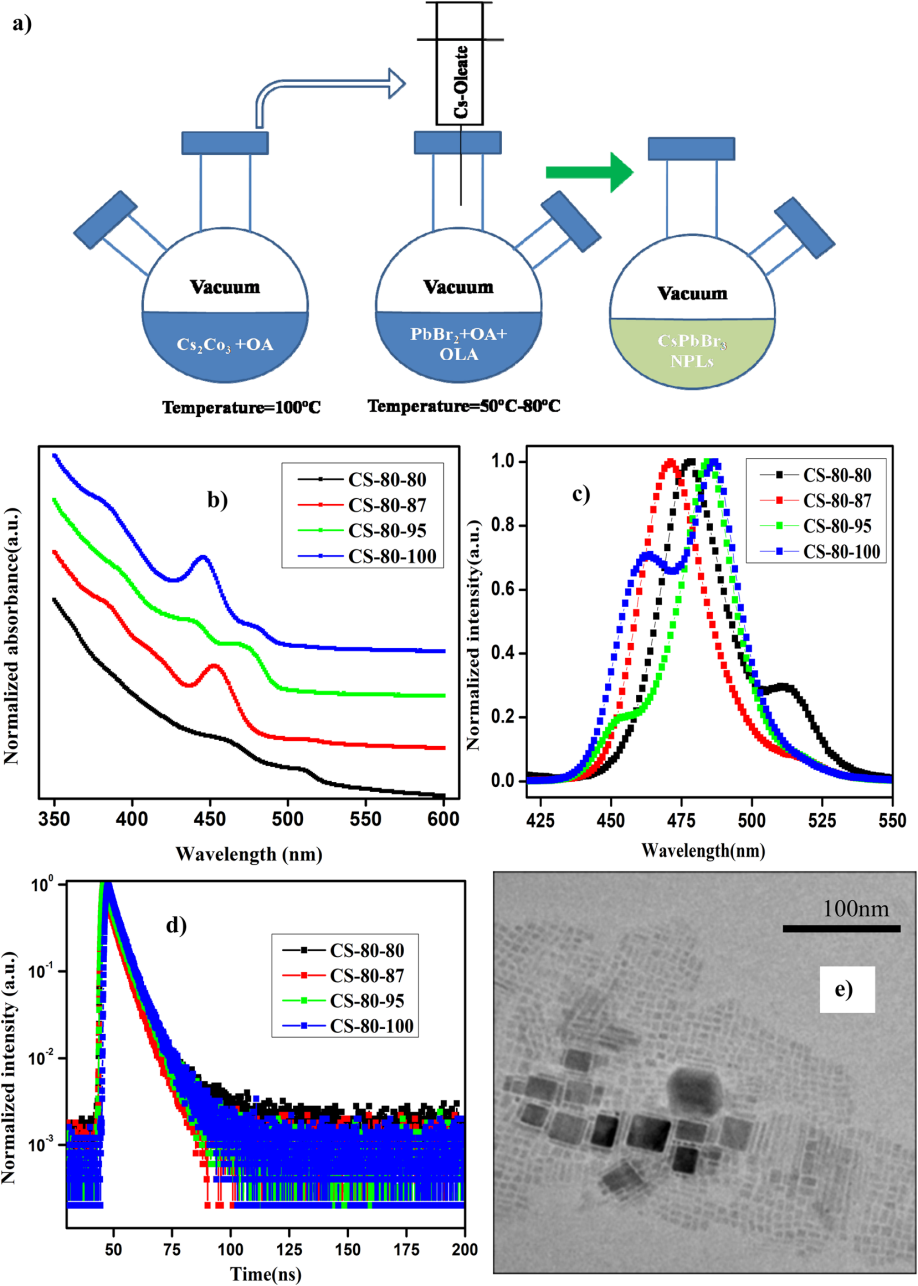
(a) Schematic diagram of the synthesis process of perovskite nanoplatelets using the vacuum-assisted low-temperature (VALT) method. (b) UV-visible absorption and (c) normalized PL spectra of the NPLs that were synthesized at 80 °C. (d) TRPL decay curves of the NPLs synthesized at 80 °C with different preheating temperatures for the PbBr_2_ precursor. (e) TEM image of the sample CS-80-95.

The details of the synthesis procedure for the perovskite NPLs can be found in ref. 47, and a schematic diagram of the synthesis process is presented in Fig. 1a. All chemicals were purchased from Sigma-Aldrich and used without any further purification.

#### 2.1.1 Preparation of Cs-oleate

Briefly, 105 mg of Cs_2_CO_3_ was dissolved in 5 mL of octadecene, and 0.5 mL of oleic acid was added to the solution. The flask was placed under a vacuum, and the temperature was raised to 100 °C.

#### 2.1.2 Synthesis and purification of NPLs

In another Schlenk flask, 69 mg of PbBr_2_ was loaded in 5 mL of octadecene. After the temperature of the flask reached 50 °C, 0.5 mL of oleic acid and 0.5 mL of oleylamine were subsequently added to it. In the next step, the temperature of the flask was maintained at the desired reaction temperature from 50–80 °C. For the pristine samples, the temperature of the flask was maintained at same reaction temperature. However, for the *in situ* passivated samples, to increase the chemical availability of Br, we heated the PbBr_2_ precursor to various higher temperatures. For example, in the case of the reaction at 80 °C, we raised the temperature of the PbBr_2_ precursor to various values, such as 80 °C, 87 °C, 95 °C, and 100 °C, in different reactions. The flask was maintained at that temperature for a few minutes in every reaction. Subsequently, the PbBr_2_ precursor was cooled to the reaction temperature *i.e.*, 80 °C. Once the flask had cooled to the desired reaction temperature, 0.4 mL of Cs-oleate was drawn up in a long syringe needle and injected swiftly into the reaction flask, which was maintained at that temperature for a few minutes. The reaction temperatures were set at 50 °C, 60 °C, 70 °C, and 80 °C, and the prepared NPLs were labeled as CS-50-50, CS-60-60, CS-70-70, and CS-80-80 respectively. In the case of samples heated to a higher temperature, the samples were designated as, *e.g.*, CS-80-87. The first number represents the reaction temperature, and the second number represents the PbBr_2_ precursor heating temperature. After the reaction was finished, the reaction mixture was cooled in an ice bath for about 20 min, and 15 mL of methyl acetate was added. The crude solution was transferred into centrifuge tubes and placed in the fridge at 4 °C for about 20 min. The NPLs were segregated at the bottom of the centrifuge tube due to the polarity of the methyl acetate. The excess ligands and unreacted precursors were separated from the crude solution. Hexane was added to the segregated solid product, which was again placed in the fridge. The Cs-oleate and Pb-oleate products were segregated at the bottom of the centrifuge tube, and the remaining supernatant was collected for further analysis. One of the crucial factors for preparing the NPLs is the unoxidized oleic acid. Since it is highly sensitive to heat, temperature, and oxygen, oleic acid can easily be oxidized under the ambient atmosphere. Since there is a highly dynamic matter on the surface of NPLs, the atoms can easily detach from the surface, which will influence the stability of the NPLs. The synthesis of NPLs with oxidized oleic acid causes the immediate degradation of CsPbBr_3_ when exposed to a polar solvent such as methyl acetate. The NPLs prepared using oxidized oleic acid easily aggregate into large-sized NCs in one to two days due to the ligand loss. Due to the ripening and aggregation of the NPLs, there is an unwanted shift in the emission peak. Therefore, we conclude that unoxidized oleic acid plays an important role in the synthesis of NPLs.

### 2.2 Sample characterization

The transmission electron microscope (TEM) images were acquired using a JEM-2100 microscope equipped with an electron gun, which was operated at 200 kV. The samples were prepared by drop-casting the highly concentrated NPL colloidal solution onto 200-mesh carbon-coated copper grids for imaging. The edge width and length of the NPLs were estimated using the software ImageJ (Fiji). The X-ray diffraction (XRD) pattern of the synthesized NPLs was determined using an X-ray diffractometer (Rigaku, MiniFlex 600) using a CuKα source. The XRD pattern was collected over a 2*θ* angle range from 3–60°. The samples for XRD were prepared by drop-casting the concentrated NPL dispersion onto a silicon wafer. The UV-visible absorption spectra of the synthesized NPLs were measured in the wavelength range of 350–600 nm using a Shimadzu UV-1800 spectrophotometer. The samples for absorbance measurements were diluted sufficiently to reduce the absorbance to less than 0.1 at a wavelength of 400 nm. The photoluminescence (PL) spectra were recorded using a Cary Eclipse fluorescence spectrometer (Agilent Technologies). We used a Jobin Yvon FluoroLog 2 spectrofluorometer to carry out the time-resolved photoluminescence (TRPL) measurements of the synthesized NPLs, which were dispersed in hexane. Here, we used a LASER diode for excitation of the samples at an excitation wavelength of 375 nm. A time-correlated single-photon counter was used to collect the photons at the peak emission of the individual samples. The PLQY of the NPLs at an excitation wavelength of 400 nm was estimated using coumarin 153. The PLQY of coumarin 153, which was dissolved in ethanol, was taken as 47% at 400 nm based on ref. 6. The absorbance of the synthesized NPLs, which were dispersed in hexane, varied from 0.01 to 0.1 to estimate the PLQY. PLQY measurements were performed on each sample, and the degradation of the PLQY values of the samples was studied for one month. Fourier-transform infrared (FTIR) spectroscopy measurements were performed on the NPLs, which were dispersed in hexane, using a Cary 630 FTIR (Agilent Technologies).

### 2.3 LED (light emitting diode) fabrication and characterization

The synthesized CS-60-80 colloidal dispersion was drop-cast onto commercially available GaN chips that can emit at *λ* = 400 nm. The samples were repeatedly coated with the colloidal dispersion several times and dried under the ambient atmosphere. The electroluminescence spectra were measured under a forward bias using a Keithley 2400 sourcemeter and a Jobin Yvon Fluorescence spectrometer.

## 3. Results and discussion

In Fig. 1b and c, we present the absorption and normalized PL spectra of the NPLs synthesized at 80 °C, for which the PbBr_2_ precursor was pre-heated to different temperatures above the reaction temperature. It can be observed in the images that multiple peaks appear in the absorption and PL spectra, which can be attributed to the formation of NCs of different sizes, such as NPLs and 3D NCs. It is evident from recent reports that multiple-peak emission in PL spectra is due to the presence of different populations of NCs that have different sizes.^18,44,48^ Hence, we presumed that the existence of multiple peaks in the absorption and PL emission originates from populations with different sizes. The NPLs that were synthesized at 80 °C (CS-80-80) exhibit absorption onset at 525 nm in the UV-visible spectrum, and another kink corresponding to the exciton appears at 470 nm (Fig. 1b, black curve). This implies the existence of two ensembles of NCs formed at this reaction temperature. The presence of ensembles of NCs of different sizes was further confirmed by the PL peaks, which were centered at 475 nm and 510 nm (Fig. 1c, black curve). The PL peak at 510 nm corresponds to green emission, and the emission below 470 nm corresponds to blue emission. The PL peak emission and absorption edge occur at the same wavelength, indicating radiative recombination from the conduction band edge. In the next case, when the PbBr_2_ precursor is heated to a higher temperature, *i.e.*, 87 °C (CS-80-87), the absorption onset appears at 510 nm, and another small kink appears at 475 nm (Fig. 1b, red curve). This implies that with the availability of Pb and Br, the growth dynamics changed, and the preferential growth of asymmetric NPLs dominated. This was further confirmed from the PL measurements, which showed the existence of two peaks centered at 470 nm and 510 nm. Additionally, the intensity of the PL peak at 510 nm is substantially reduced compared to that of the pristine samples. In the next case, when the PbBr_2_ precursor temperature is raised to 95 °C (CS-80-95), the absorption kink starts at 480 nm, and the absorption peak at 510 nm completely vanishes. The PL peak is now centered at 480 nm, which lies between the emission peaks of the previous sample, *i.e.*, 470 and 510 nm. This implies that the excess Br that is soluble at higher temperatures changes the growth dynamics and significantly affects the size and shape of the NCs. Additionally, another absorption onset appears at 450 nm (Fig. 1b, green curve), corresponding to the low-dimensional NPLs. This implies that the chemical availability of Br changed the formation dynamics of the NPLs. It also indicates that the formation energy of NPLs that can emit at 460 nm is low. The NPLs that can emit at 460 nm might correspond to three monolayers (3 MLs).^21^ With further increasing the temperature of PbBr_2_ to 100 °C, the absorption peak (Fig. 1b, blue curve) centered at 460 nm becomes more intense, and the PL peak intensity increases as well (Fig. 1c, blue curve). It is worth noting that with increasing the PbBr_2_ precursor temperature, the relative intensity of the blue peak increases, and that of the green peak decreases. Bernhard *et al.* demonstrated that control over the thickness of NPLs can be achieved by regulating the ratio of Cs/Pb in addition to the temperature.^21^ Wu *et al.* proved that the addition of HBr to the PbBr_2_ precursor solvent helps in the formation of PbBr_6_^4−^ octahedra, which will reduce the trap density and improve the PL characteristics.^26^ Gao *et al.* demonstrated a novel colloidal method for the synthesis of ultrathin nanoribbons.^5^ They showed that the anisotropic growth of NCs and the formation of platelet structures can be facilitated by the suppression of the reactivity of the Cs ions. Furthermore, they showed that the number of monolayers can be regulated by the amount of HBr added to the precursor. These studies reveal that the *in situ* addition of Br would improve the defect-free formation of the PbBr_6_^4−^ octahedra and reduce the defect states, helping to improve the PL characteristics. Wen *et al.* further elucidated that the chemical availability of Br dictates the growth mechanism.^40^ The growth of NCs starts with the formation of the CsBr complex, and these CsBr seeds further react with the Br and form the intermediate product Cs_4_PbBr_6_. With further reaction with Br, finally, the compound CsPbBr_3_ is formed. Akkerman *et al.* showed that the thickness of the NPLs can be controlled up to a monolayer by regulating the amount of HBr during the synthesis.^24^ They showed that the acidity of HBr increases the protonation of oleylamine on the surface of the NPLs. These protonated oleylamine ligands can compete with the Cs^+^ ions and replace them on the surface of the platelets. This will increase the growth of the NPLs along the lateral direction and slow the growth in the vertical direction.^5,24,25^ These reports indicate that the availability of Br and the ratio of Cs/Br play an important role in regulating the number of monolayers in NPLs. In this work, we further confirmed that the availability of Br during the reaction can be improved by preheating the PbBr_2_ precursor to a higher temperature. The availability of Br during the reaction changes the growth dynamics and promotes the formation of NPLs with fewer defects, which can enhance the PL characteristics. Photographic images under white light and UV-light of the as-synthesized samples that were prepared at 80 °C under different conditions are presented in ESI Fig. S1.† Photographic images under white light and UV light of the same samples after being stored under the ambient atmosphere for a month are presented in ESI Fig. S2.†

The PLQY values of the samples that were synthesized at a temperature of 80 °C with different preheating temperatures for the PbBr_2_ precursor are presented in Table 1. The PLQY of the CS-80-80 sample (as-synthesized) is about 10%. This value is quite low due to the presence of impurities such as Cs-oleate and Pb-oleate, which will act as trap states for charge carrier recombination. As the impurities became segregated at the bottom of the vial, the PLQY value later reached 30%. These estimated PLQY values for four-monolayer NPLs are consistent with the results published in ref. 21 for non-passivated samples of four-monolayer-thick NPLs. In the case of the CS-80-87 sample, the PLQY is about 20–25%, and remains stable for a month. However, this PLQY value is relatively low compared to that of the CS-80-80 sample. The PL spectrum of the CS-80-80 samples gives a clear indication of the presence of two types of ensembles: one ensemble consists of large-sized NCs and the other comprises weakly confined NPLs. Since the NC ensembles are 3D in shape, the number of atoms on the surface is relatively lower compared to that for the NPLs. Hence, there are fewer undercoordinated atoms, which will minimize the recombination rate; consequently, these will exhibit relatively high PLQY. However, in the case of the CS-80-87 sample, the PL peak at 510 nm is substantially low. This implies that the formation of NPLs is more predominant at this temperature. However, the NPLs inherently contain a greater number of surface defects due to their high surface-to-volume ratio. Hence, the overall PLQY value of CS-80-87 is relatively low, since the sample comprises mostly NPLs. The PL intensity as a function of the wavelength for different absorption values at an excitation wavelength of 400 nm for the sample CS-80-95 is presented in ESI Fig. S3a.† The integrated PL intensity *vs.* absorbance for the reference sample coumarin 153 and CS-80-95 are presented in ESI Fig. S3b.† On the other hand, the PLQY value of the CS-80-95 sample reaches 100% and remains stable for a month. This implies that when the PbBr_2_ precursor solution is heated to a higher temperature in the presence of ligands, the chemical availability of Br dictates the preferential growth of NPLs with fewer defects. Due to the passivation of these surface traps, recombination of the charge carriers is minimal, which will enhance the PLQY substantially. With further increasing the PbBr_2_ precursor temperature to 100 °C, the excess Br initiates the formation of the NPLs that can emit at 460 nm. It is evident from the PL spectrum of CS-80-100 that the sample consists of two types of ensembles: one ensemble consists of weakly confined NPLs that can emit at 475 nm, and the other ensemble comprises confined NPLs that can emit at 460 nm. In practice, these strongly confined ensembles contain a greater number of surface traps, and hence the overall PLQY of the sample CS-80-100 is relatively low compared to that of CS-80-95. Kang *et al.* calculated the formation energies of the defects in CsPbBr_3_ crystals from the first principles. The formation energy of the defects under Br-rich conditions is small compared to the formation energy of the defects in a Br-poor environment. Hence, Br-rich conditions are not good for the growth of defect-free crystals, and a moderate amount of Br is sufficient for the growth of crystals with fewer defects.^36,37^

**Table 1:** PLQY values of the samples that were synthesized at 80 °C with preheating of the PbBr_2_ precursor to different higher temperatures

Sample name	As-synthesized	1 week	2 weeks	1 month
CS-80-80	10.84	31.70	28.40	36.78
CS-80-87	20.66	27.01	26.04	26.41
CS-80-95	95.24	96.49	99.67	97.41
CS-80-100	44.60	39.78	42.24	37.49

These synthesized NPLs usually exhibit a band-tail emission, which can be assigned to trap states. The asymmetric PL emission in the long-wavelength region is suppressed for the NPLs, implying a reduced defect density.^23,26^ To further understand the trap-related mechanism in NCs, the Urbach energy (*E*_U_) was estimated. Smaller *E*_U_ values represent high crystallinity of the NCs. This can be calculated by plotting the absorption coefficient (*α*) as a function of the photon energy (*E*). The Urbach tail is longer for the semiconductors, which have a high degree of disorder, and is negligible for highly crystalline samples. The *E*_U_ value can be extracted by fitting the following expression to the *α vs. E* graph.1
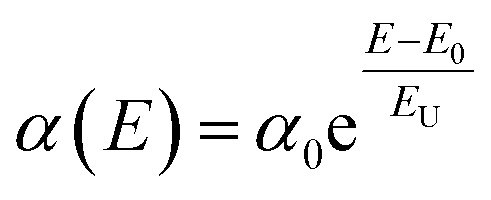


The calculated *E*_U_ value of the pristine CsPbBr_3_ NPLs (CS-80-80) is about 101 meV, which implies that a large amount of disorder is present in the NCs. However, in the case of the CS-80-87 sample, the *E*_U_ value decreases to 80 meV. Similarly, in the case of CS-80-95, its value is further reduced to 48.5 meV. These results confirm that the excess Br during the synthesis process allows the growth of NPLs with fewer defects. On the other hand, in the case of the CS-80-100 sample, the *E*_U_ value again increases to 66.6 meV. This is due to the formation of NPLs, which have more surface defects. It implies that the excess Br will induce additional trap states and might act as nonradiative recombination centers for the charge carriers.^7,26^

To further evaluate the effect of traps and obtain a better understanding of the charge transport phenomenon on the synthesized NPLs, we performed TRPL decay measurements. The TRPL decay curves of the synthesized NPLs at 80 °C are presented in Fig. 1d. The excitation wavelength is 375 nm. The decay curve was fitted using the following double exponential function:

2 *y* = *y*_0_ + *C*_1_e^−(*t*/*τ*_1_)^ + *C*_2_e^−(*t*/*τ*_2_)^

where *y*_0_, *C*_1_, and *C*_2_ are constants and *τ*_1_ and *τ*_2_ are the double exponential time constants. The time constants are extracted by fitting the above equation to the TRPL decay curves.^23^ The average lifetime of the charge carriers is estimated using the following formula:3
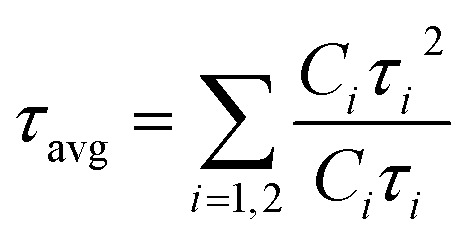


The distribution coefficients *C*_1_ and *C*_2_ and the time constants for the samples that were prepared at 80 °C are presented in ESI Table S1.† The average lifetime of the charge carriers for the pristine samples (CS-80-80) is 3.26 ns. The lifetime for the CS-80-87 samples is 2.49 ns, which is relatively shorter than that of the pristine samples. In the case of the pristine samples, the presence of some large-sized NCs is evident from the PL and absorption spectra (510 nm emission). These cubic crystals have few atoms on the surface; hence, the recombination is minimal, and thus the lifetime will be longer. However, in the case of the CS-80-87 samples, it is evident from the PL emission spectrum that the formation of NPLs is predominant over that of large-sized NCs. Since the NPLs have a greater surface-to-volume ratio, the undercoordinated atoms on the surface of the NPLs form the trap states. These trap states will act as recombination centers, and hence, the average lifetime of the charge carriers is reduced compared to that of the pristine samples. In the case of the CS-80-95 samples, the lifetime of the charge carriers is relatively high (4 ns) due to the formation of NPLs with fewer defects. The formation of highly crystalline NPLs is due to availability of Br, which reduces the formation of defects. In the case of the CS-80-100 sample, the lifetime of the charge carriers decreases due to the formation of the NPLs, which have peak emission at 460 nm. Since these strongly confined NPLs are highly prone to surface defects, the charge carrier lifetime will be shorter.^26^ These results convey that the heat treatment of the PbBr_2_ precursor solution to a high temperature improves the solubility and enhances the chemical availability of Br.^26^ This excess Br remarkably reduces the undercoordinated atoms on the surface. Consequently, the nonradiative recombination of charge carriers will be minimal, and hence, the lifetime of the charge carriers will be enhanced.

To further confirm the mechanism of the formation of the NPLs, we characterized the samples that were synthesized at 80 °C with different preheating temperatures for the precursor PbBr_2_ using XRD. In ESI Fig. S5a,† we present the XRD pattern of the CS-80-80 sample. In this pattern, the peaks appeared at angles of 15.2°, 18.2°, 21.8°, 25.8°, 28.6°, and 39.4°. The peaks at 15.2°, 21.8°, and 28.6° correspond to the (100), (110), and (200) planes of the CsPbBr_3_ crystal structure.^24^ Additionally, the peak that appears at 28.6° is very wide and has a long tail, which represents the formation of a broad size distribution of NCs. The peaks that appear at 18.2°, 25.8°, 35.6°, and 39.4° correspond to the formation of the Cs-rich compound Cs_4_PbBr_6_.^49^ The formation of the intermediate compound Cs_4_PbBr_6_ indicates the unavailability of PbBr_2_ at the reaction temperature due to its limited solubility.^40^ To increase the solubility of PbBr_2_, we heated the PbBr_2_ precursor to various higher temperatures, namely, 87 °C, 95 °C, and 100 °C, in different reactions. In ESI Fig. S5b,† we present the XRD pattern of the sample CS-80-87. It can be observed from this pattern that the intensity of the peaks at 25.8° and 39.4° vanishes completely, which signals that the formation of the intermediate compound Cs_4_PbBr_6_ ceases. On the other hand, the intensity of the peak at 28.6° increases, which indicates the growth of NPLs with a narrow size distribution. On the other hand, the peak that corresponds to the (200) plane of the NPLs is shifted to a lower value of 28.2°, which corresponds to the same plane that was observed in the bulk NCs at 30.2°.^24^ The interplanar spacing of the (200) planes was estimated to be 0.312 nm, which is in good agreement with the value obtained from the TEM images, as discussed below. Using the most intense diffraction peak, which corresponds to the (200) plane, a unit cell size of 0.624 nm was estimated. The formation of a high-intensity (200) plane peak gives a clear indication of the formation of the NPLs with a preferred orientation whose axis is parallel to the normal of the substrate surface.^21^ According to the DFT calculations of Dong *et al.*, the surface energy of the (010) plane is less than the surface energy of the (110) and (111) planes.^44^ This implies that the adatoms during the formation of the crystals will preferably occupy the (010) plane rather than other planes. Similarly, when the PbBr_2_ precursor temperature is raised to 95 °C, the intensity of the peaks at 25.8°, 35.6°, and 39.4° completely vanishes. This indicates that the formation of CsPbBr_3_ is predominant when the chemical availability of Pb and Br is improved. In ESI Fig. S5c,† we present the XRD pattern of the CS-80-95 sample. In this pattern, the peak at 28.6° is split into two peaks appearing at 28.2° and 27.8°, indicating the formation of two ensembles of NPLs. The peak at 27.8° corresponds to the ensemble of NPLs that show PL emission at 460 nm. Similarly, the peak at 28.2° corresponds to the ensemble of NPLs that exhibit PL emission at 475 nm. Furthermore, the intensity of the peak at 27.8° is relatively higher than that of the peak at 28.2°. This reveals the formation of the ensemble of NPLs with PL emission at 475 nm is more predominant than the formation of the ensemble of the NPLs whose PL emission is observed at 460 nm^5^. In ESI Fig. S5d,† we present the XRD pattern of the sample CS-80-100. In this pattern, the peak at 27.8° becomes more pronounced, and the peak intensity at 28.2° is reduced. This reveals that the formation of the ensemble of NPLs that can emit 460 nm is more predominant than the formation of the ensemble of NPLs whose PL emission observed is at 475 nm. It was concluded that increasing the amount of Br in the precursor solution increases the formation of NPLs of lower dimensions. These results are in good agreement with the PL properties, as discussed above.

To further confirm our hypothesis, we prepared samples of PbBr_2_ dissolved in octadecene in the presence of oleic acid and oleylamine. The PbBr_2_ precursor was heated to a temperatures of 87 °C, 95 °C, and 100 °C, which are higher than that for the preparation of the NCs (80 °C). Photographic images of the samples under white light and UV light are presented in ESI Fig. S7.† It can be observed from the image that as the temperature of the PbBr_2_ precursor is increased, its colour changes from transparent to pale yellow. This implies that as the precursor temperature is raised, a greater amount of Br is soluble, which will change the color of the solution from transparent to pale yellow. Similarly, when the same samples were exposed to UV light (ESI Fig. S8†), the contrast in the solution changed gradually from violet to dark blue as the temperature of the PbBr_2_ precursor is changed from 80 to 100 °C. This implies that as the precursor temperature is increased, the excess Br that is soluble helps in the formation of PbBr_6_^4−^ octahedra.^26^ Hence, due to the formation of the octahedra, the emission takes place in the blue region. With increasing the temperature of the precursor, the octahedra are formed with fewer defects, and hence, the blue emission becomes stronger. These results were further evidenced by the UV-visible absorption data that are presented in ESI Fig. S7.† It is evident from the spectra that as the precursor temperature increases, the absorption in the wavelength region of 350–400 nm increases; additionally, the kink at 365 nm represents the formation of the PbBr_6_^4−^ octahedra.^26^

TEM was employed to determine the size and morphology of the synthesized NCs. The main limitation of TEM for the analysis of NPLs is that they are sensitive to the incident electron beam, and their atomic structure can be easily altered by the electron beam.^50^ In Fig. 1e, we present a TEM image of the sample CS-80-95. It can be seen from the image that some NPLs are lying flat, and a few of them are stacked face-to-face with each other. The thickness of the NPLs was estimated to be 2.4 ± 0.3 nm, which might correspond to four monolayers. Similarly, the separation between the NPLs is about 1.4 nm, which corresponds to the ligands.^21^ Additionally, the length and width of the NPLs are about 4 nm and 6 nm, respectively, and we can ascertain that these NPLs are four monolayers thick.^21^ In Fig. S4,† we present the TEM image of a single NPL. The separation between the planes was estimated to be 0.3 nm, which is in close agreement with the value estimated from XRD, which corresponds to the (200) plane. Additionally, some thicker NPLs are present, which are formed due to poor ligand stabilization. These large and thick sheets are formed by the attachment of single NPLs that have the same crystallographic orientation under ligand-destabilized conditions.^19^

**Fig. 2:**
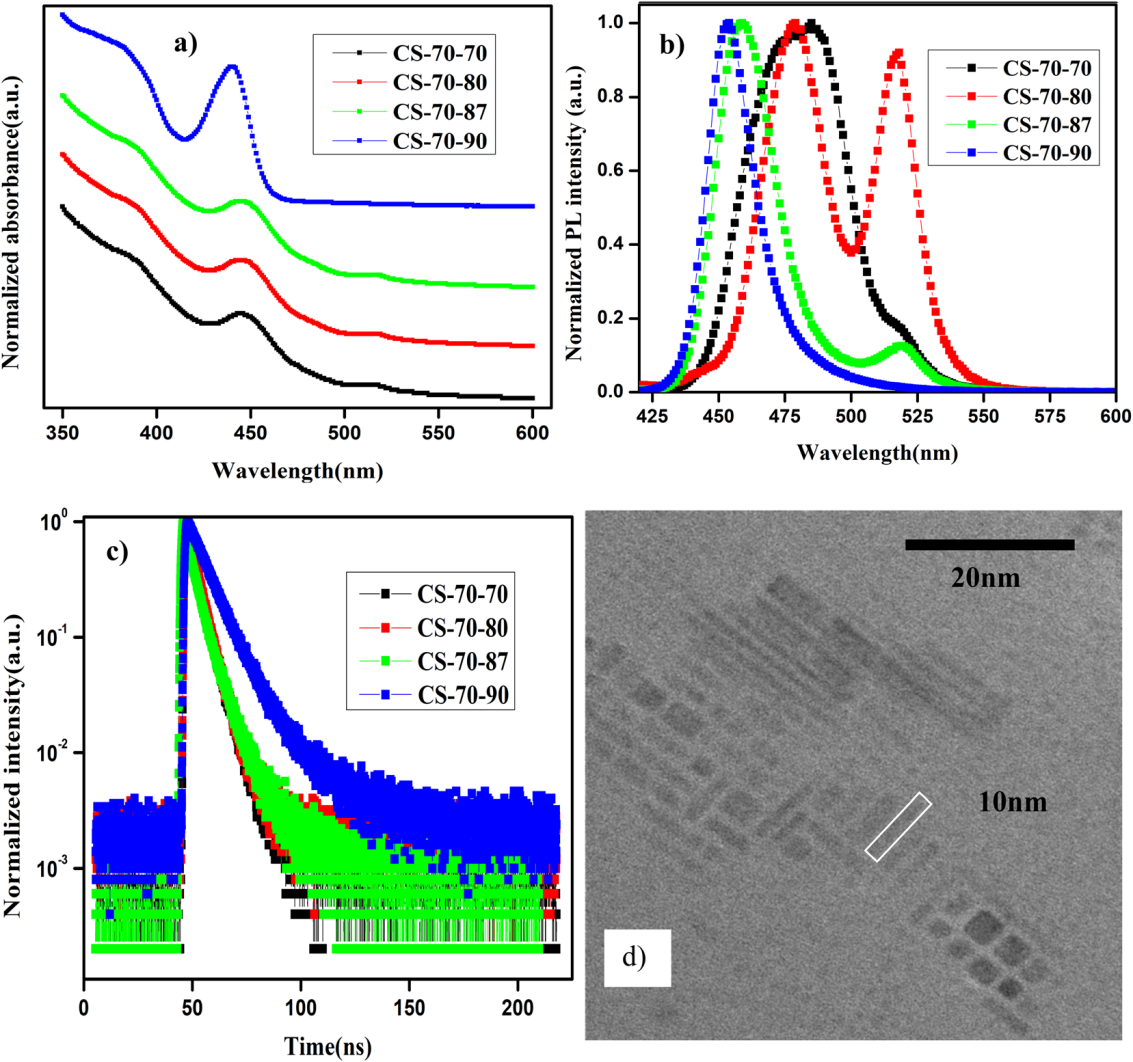
(a) UV-visible absorption and (b) normalized PL spectra of the NPLs that were synthesized at 70 °C. (c) TRPL decay curves of the NPLs synthesized at 70 °C that were prepared under different conditions. (d) TEM image of the sample CS-70-90.

In Fig. 2a and b, we present the UV-visible absorption and normalized PL spectra of the NPLs that were synthesized at 70 °C, for which the PbBr_2_ precursor was heated to different temperatures. In the case of the CS-70-70 samples, the absorption onset starts at 520 nm, and another kink is observed at 450 nm (Fig. 2a, black curve). In the same way, the PL emission extends over a broad range from 440 nm to 520 nm (Fig. 2b, black curve). This implies that at this reaction temperature, NCs of different sizes are formed with an enormous amount of defects, and hence, the emission extends over a wide range. When the PbBr_2_ precursor temperature is increased to 80 °C (*i.e.*, CS-70-80), two PL peaks are observed in the PL spectrum, which are centered at 475 nm and 520 nm (Fig. 2b, red curve). These could be due to some NPLs becoming fused into large-size NCs due to poor ligand stabilization.^19^ In the next case, when the temperature of the PbBr_2_ precursor is raised to 87 °C (CS-70-87), the PL emission is shifted to 455 nm, and the small peak is still present at 520 nm (green curve). With further increasing the PbBr_2_ precursor temperature to 90 °C, the PL emission is shifted to 463 nm (Fig. 2b, blue curve). In this case, the emission peak lies between 455 and 520 nm. This implies that when the PbBr_2_ precursor temperature is raised to 90 °C, the excess Br that is soluble helps in the formation of NPLs with fewer defects. Furthermore, in the case of the CS-70-90 samples, a step-like increase in absorption and a small Stokes shift of 10 nm are observed in the absorption spectrum (Fig. 2a, blue curve). The step-like increase in the absorption spectrum resembles the clear exciton peak for a 2D semiconductor. This implies that the binding energy of the exciton also increased, since the thermal energy that is available at room temperature is not able to dissociate the exciton.^21,35^ Photographic images under white light and UV-light of the as-synthesized samples that were prepared at 70 °C under different preheating temperatures for the PbBr_2_ precursor are presented in ESI Fig. S9.† The photographic images under white and UV light of the same samples after being stored under the ambient atmosphere for a month are presented in ESI Fig. S10.†

The PLQY values of the samples that were synthesized at a temperature of 70 °C are presented in Table 2. The PLQY value of the pristine samples (CS-70-70) with peak emission at 475 nm is about 30%, and it is stable for a month. In the case of the CS-70-80 samples, two emission peaks centered at 475 nm and 520 nm were observed. However, the yield of the NPLs that emit at 475 nm is quite low, and hence, the overall PLQY value is lower. Over time, all the NCs that emit at 520 nm settled at the bottom of the vial, and the PLQY of the NPLs that can emit at 475 nm rose to 70% and remained stability for a period of one month. In the next case, when the PbBr_2_ precursor temperature is raised to 87 °C, the PL peak is shifted to 455 nm and the PL emission peak at 520 nm is almost suppressed. However, the PLQY value is 30%, which is relatively low. This was because the NPLs that are formed at this temperature contain more defects due to the high surface-to-volume ratio. Hence, the overall PLQY value decreases. With further increasing the temperature of the PbBr_2_ precursor to 90 °C and increasing the stirring time, more Br becomes soluble, and hence NPLs with fewer defects will be formed. Hence, the PLQY of these samples reached 70%. These samples can retain 85% of their initial PLQY value even after one month of being exposed to the ambient atmosphere. In ESI Fig. S13a,† we present the PL intensity of the sample CS-70-90 at an excitation wavelength of 400 nm for different absorbances. The integrated PL intensity as a function of absorbance for CS-60-80 and the reference sample coumarin 153 are presented in ESI Fig. S13b.† However, the PL emission of CS-70-90 is asymmetric and has a long tail, which implies the formation of a broad distribution of NCs. With further increasing the PbBr_2_ precursor temperature above 90 °C, the PLQY decreases.

**Table 2:** PLQY values of the samples that were synthesized at 70 °C with preheating of the PbBr_2_ precursor to different higher temperatures

Sample name	As-synthesized	1 week	2 weeks	1 month
CS-70-70	31.48	37.69	28.66	33.08
CS-70-80	16.55	70.92	68.99	62.05
CS-70-87	14.07	36.28	27.29	35.91
CS-70-90	70.51	56.32	51.59	58.27

In Fig. 2c, we present the TRPL decay curves of the samples that were prepared at 70 °C under different conditions. The TRPL decay curves are well fitted by eqn (2), and the average lifetime of the charge carriers was estimated by using eqn (3). The time constants, distribution coefficients, and the average lifetime of the charge carriers are presented in ESI Table S2.† The average charge carrier lifetime of the pristine samples (CS-70-70) is about 3.58 ns. In the case of the CS-70-80 samples, the average lifetime decreases to 2.58 ns. The shorter lifetime of the charge carriers is due to the formation of NPLs that contain an enormous amount of defects. Furthermore, it is evident from the PL measurements that two types of NCs with different sizes are formed. Since the NPLs contain more defects, the overall charge carrier lifetime decreases. Similarly, in the case of the CS-70-87 samples, the lifetime of the charge carriers increases to 4.67 ns, which is longer than the lifetime of the pristine samples. The excess Br that is soluble at this temperature during the reaction forms the NPLs with fewer defects, and hence, the overall charge carrier lifetime increases. In the next case, when the PbBr_2_ precursor temperature was further raised to 90 °C, the average lifetime increased to 9.69 ns.

The *E*_U_ value was extracted using formula (1) by plotting the curve of *α vs. E*. The *E*_U_ value decreases gradually from 133 meV to 44 meV as the PbBr_2_ precursor temperature increases from 70 to 90 °C. This further confirms that increasing the PbBr_2_ precursor temperature to higher values increases the solubility and the availability of Br during the reaction, helping to form NPLs with fewer defects.

In ESI Fig. S11a–d,† we present the XRD patterns of the samples that were synthesized at 70 °C with raising the PbBr_2_ precursor temperature to different values. In ESI Fig. S11a,† the XRD pattern of CS-70-70 shows peaks at 18.2°, 21.7°, 25.7°, 28.6°, and 39.2°. The peaks at 18.2°, 25.7°, and 39.2° that are marked with an asterisk represent the formation of the intermediate compound Cs_4_PbBr_6_.^49^ Similarly, the peaks that appear at 21.7° and 28.6° represent the formation of the (110) and (200) planes of CsPbBr_3_.^5,23^ Additionally, the peak that appears at 28.6° is very broad with a long tail, which represents the formation of NCs of different sizes, which is consistent with the PL data in which the emission ranges from 450 nm to 520 nm (Fig. 2b). In ESI Fig. S11b,† we present the XRD pattern of the NPLs of the sample CS-70-80. In this pattern, one can observe that the peak of 28.6° becomes slightly narrower and less intense. This indicates that the NCs that are formed have poor crystallinity and a broad size distribution. Furthermore, it exhibits two peaks at 28.5° and 29.2°, which represent the formation of the two sets of ensembles. The additional peaks at other angles corresponding to the formation of the intermediate compound Cs_4_PbBr_6_ completely vanish. In ESI Fig. S11c,† we present the XRD pattern of the sample CS-70-87. In this pattern, the peak at 28.5° that corresponds to the (200) plane of the NPLs is split into two peaks, one at 28.2° and another at 28.5°. This implies the formation of two sets of ensembles: one ensemble corresponding to the formation of NPLs, and another ensemble corresponding to the formation of large-sized NCs. These results are in good agreement with the PL data. In the PL emission spectra of the same sample CS-70-87 (Fig. 2b, green curve), two peaks were observed that correspond to two different sizes. Similarly, in the case of the CS-70-90 samples, the XRD pattern exhibits an intense peak at 28.2°, and the remaining peaks are relatively low intensity formation of NPLs, which implies the formation of large-sized NCs ceases.

In Fig. 2d, we present the TEM image of the sample CS-70-90. The width of the NPLs was estimated to be 2 nm, and the length of the NPLs is 10 nm. The separation between the NPLs was estimated to be 1.4 nm due to the presence of the ligands on the surface of the NPLs.^5^ This implies that when the reaction temperature is reduced to 70 °C, asymmetric growth of the NPLs takes place.^19^ Additionally, it can be observed from these images that NPLs of different sizes are formed, which might be the reason for the presence of the long tail in the PL emission spectrum.

**Fig. 3:**
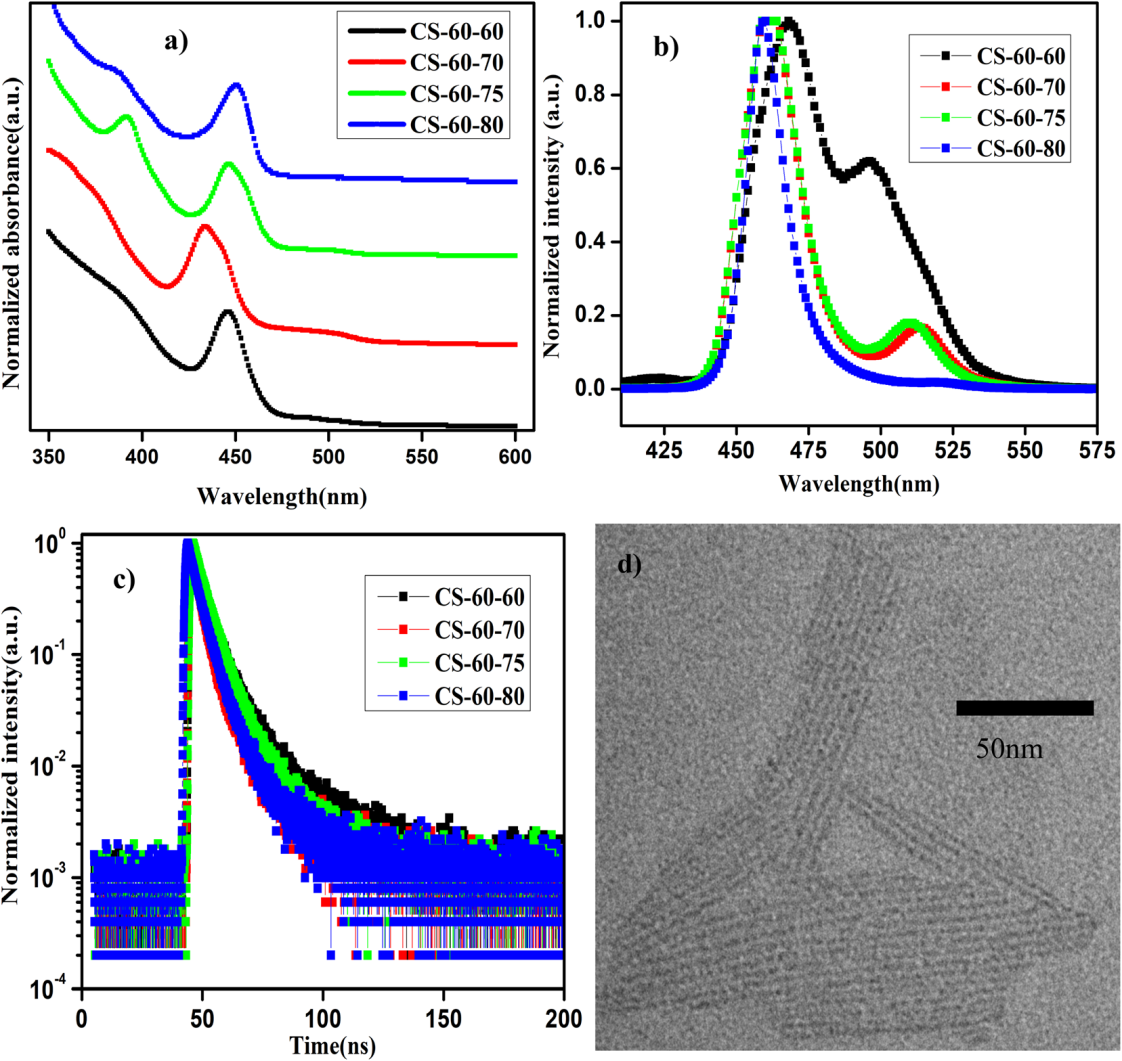
(a) UV-visible absorption and (b) normalized PL emission spectra of the NPLs synthesized at 60 °C with different PbBr_2_ precursor temperatures. (c) TRPL decay curves for the NPLs synthesized at 60 °C under different conditions. (d) TEM image of the sample CS-60-80.

In Fig. 3a and b, we present the UV-visible absorption and normalized PL spectra of the NPLs that were prepared at 60 °C with different PbBr_2_ precursor heating temperatures. It can be observed from the absorption spectra that all the samples show a step-like increase in the absorbance at 460 nm, which is due to the strong quantum confinement of the charge carriers. This implies that as the reaction temperature is reduced, asymmetric growth of NPLs with a thickness of a few monolayers takes place. When the thickness of the NPL is reduced below the Bohr radius, strong quantum confinement of the charge carriers takes place. Due to the quantum confinement of the charge carriers in the NPLs, the absorption changes from a continuous spectrum to a step-like one, which resembles the energy levels of a 2D semiconductor, along with a sharp excitonic peak.^21^ Furthermore, in the case of the pristine samples (CS-60-60), the absorption spectrum has two kinks that appear at 520 nm and 480 nm (Fig. 3a, black curve) and the corresponding PL emission is centered at 500 nm and 470 nm (Fig. 3b, black curve). In the case of the CS-60-70 samples, two kinks appeared in the absorption spectrum, one at 510 nm and the other one at 460 nm (Fig. 3a, red curve), and the corresponding PL peaks were observed at 510 nm and 460 nm (Fig. 3b, red curve), but the PL peak intensity at 510 nm was reduced substantially. This implies that the excess Br that is available induces the formation of strongly confined NPLs and suppresses the formation of large-sized NCs. Additionally, the large Stokes shift of 20 nm implies an enormous number of defects due to the limited availability of Br. In the next case, when the PbBr_2_ precursor temperature is raised to 75 °C, the Stokes shift of 10 nm is relatively smaller than that of the CS-60-70 samples (Fig. 3a, green curve).

Additionally, the PL peak at 460 nm remains the same, but the PL peak of the larger NCs is blue-shifted to 505 nm (Fig. 3b, green curve). With further increasing the PbBr_2_ precursor temperature to 80 °C, the PL peak became centered at 455 nm, but the full width at half maximum (FWHM) of the synthesized NPLs was reduced to 10 nm. This implies that the excess Br that is soluble at this temperature helps to form PbBr_6_^4−^ octahedra without Br defects. In this case, the Stokes shift also decreases to 10 nm, which indicates the high crystallinity of the synthesized NPLs. Photographic images under white light and UV-light of the as-synthesized samples that were prepared at 60 °C under different conditions are presented in ESI Fig. S14.† The photographic images under white light and UV light of the same samples after being stored under the ambient atmosphere for a month are presented in ESI Fig. S15.†

In Table 3, we present the PLQY values of the NPLs that were synthesized at 60 °C using different preheating temperatures for the PbBr_2_ precursor. The PLQY value of the pristine sample is about 20%, which is quite low for the three-monolayer perovskite NPLs. However, the PLQY value is consistent with the reported values published in ref. 21 for the non-passivated NPLs. These low PLQY values are due to the exceptionally high surface-to-volume ratio of NPLs, which makes them prone to more surface defects. In the case of the CS-60-70 samples, the PLQY value of the as-synthesized samples is about 48%, and rises to 80% with time due to the rearrangement of the atoms. These samples retain 90% of their initial PLQY value even after one month of exposure to the ambient atmosphere. In the case of CS-60-75, the PLQY value increases to 87%; this initial value is maintained for up to two weeks and finally drops to 40% after one month. Similarly, when the PbBr_2_ precursor reaction temperature is increased to 80 °C, the PLQY value reaches up to 80% and retains 80% of its initial PLQY value even after a month. The PL intensity as a function of wavelength for the sample CS-60-80 at an excitation wavelength of 400 nm for different values of absorption is presented in ESI Fig. S16a.† The integrated PL intensity as a function of absorbance for CS-60-80 and the reference sample coumarin 153 are presented in ESI Fig. S16b.† In ESI Fig. S17,† we present the PL intensity *vs.* wavelength for the CS-60-80 samples for a period of one month. It can be observed from the figure that the PL peak does not change its position, and that no peaks at higher wavelengths corresponding to ripening or aggregation of the NPLs into larger NCs are observed, even though they were exposed to the ambient atmosphere. This indicates that these NPLs have excellent stability and do not exhibit aggregation or ripening.

**Table 3:** PLQY values of the samples that were synthesized at 60 °C with preheating of the PbBr_2_ precursor to different higher temperatures

Sample name	As-synthesized	1 week	2 weeks	1 month
CS-60-60	20.31	21.72	19.66	16.02
CS-60-70	47.96	82.74	76.39	69.32
CS-60-75	87.90	64.56	69.54	40.04
CS-60-80	79.97	92.14	74.58	59.80

To further confirm the enhancement in the PLQY, we performed TRPL measurements on these samples. The distribution coefficients and the short- and long-lifetime components of the charge carriers were extracted by fitting the data to eqn (2), the average charge carrier lifetime was calculated using eqn (3), and the results are presented in ESI Table S3.† The charge carrier lifetime of the pristine samples is 4.89 ns. In the case of the CS-60-70 samples, the average charge carrier lifetime is reduced to 3.34 ns. In the case of the CS-60-75 samples, the average charge carrier lifetime increases slightly to 4.73 ns. Similarly, in the case of the CS-60-80 samples, the average lifetime is enhanced to 4.08 ns. To further evaluate the trap density, we calculated the *E*_U_ of the synthesized NPLs using eqn (1), and the value of *E*_U_ for the pristine samples was estimated to be 60 meV. In the case of the CS-60-70 samples, the *E*_U_ value rises to 73 meV, since most of the formed crystals are NPLs. With further increasing the precursor temperature, the *E*_U_ value decreases to a lower value of 49.6 meV. Similarly, the *E*_U_ value for CS-60-80 samples is 37 meV, which is much smaller than that of the pristine samples. These results confirm that preheating the PbBr_2_ precursor solution to a higher temperature reduces the defect density.

In ESI Fig. S18a–d,† we present the XRD patterns of the NPLs synthesized at 60 °C with different preheating temperatures for the PbBr_2_ precursor. In the XRD pattern of sample CS-60-60, a broad peak appears at 28.6°, which corresponds to the (200) plane of the CsPbBr_3_ NCs (Fig. S18a†). This implies a wide distribution of the size of the NCs, which can emit over a range of wavelengths from 450 nm to 520 nm. Similarly, in the case of CS-60-70, the most intense peak appears at 28.6°, which represents the formation of NCs with fewer defects (ESI Fig. S18b†). In the case of the CS-60-75 sample, in addition to the high-intensity peak at 28.6°, a few low-intensity peaks are present at small angles, which might correspond to the stacking of the NPLs (ESI Fig. S18c†). The small-angle XRD pattern corresponding to the stacking of the NPLs is presented in ESI Fig. S19.† Furthermore, the PL peak also appears at 460 nm for three-monolayer perovskite, which is consistent with the results published in ref. 26. This implies that as more ligands are present on the surface due to the formation of defect-free octahedra, they start to interact with each other. Due to this interaction, they form an ordered lamellar structure.^19,23^ The small-angle diffraction peaks correspond to (0 0 2*l*) reflections. The periodicity of the peaks is about 2°, which gives a stacking distance of 4.4 nm. These results are in close agreement with the data that is revealed from the TEM images, as discussed below. Similarly, in the case of the CS-60-80 samples, in addition to the high-intensity peak at 28.6°, another low-intensity peak appears at 6.5° (marked with a hash symbol in ESI Fig. S18d†) corresponding to a spacing of about 1.35 ± 0.25 nm between the NPLs.^26^ A magnified image of the XRD pattern for the sample CS-60-80 at low angles is presented in ESI Fig. S19.† Furthermore, the broadening of the peak is about 1°, which implies that the spacing between the NPLs is not constant and varies about 0.5 nm between the NPLs in the lamellar structures, as shown in the TEM images below. On the other hand, an additional peak, which is marked with an asterisk, is present in the XRD pattern in ESI Fig. 18d,† which can be ascribed to the formation of the non-emissive Cs-rich compound Cs_4_PbBr_6_.^49,51^ Since these NPLs have an inherently high surface-to-volume ratio, their surface energy will be larger.^38^ Since these NPLs consist of highly dynamic matter on the surface, detachment of atoms from the surface can easily occur with exposure to the ambient atmosphere. Consequently, the Br atoms are lost from the perovskite structure, which leads to the formation of the Cs-rich compound Cs_4_PbBr_6_.^4,21,47,52^

In Fig. 3d, we present the TEM image of the NPLs of the sample CS-60-80. These perovskite NPLs show a clear tendency to self-assemble into lamellar structures. Due to the strong ligand interaction, they are stacked into columnar structures.^19^ The formation of these lamellae suggests the asymmetric growth of the NPLs. The length of the NPLs is estimated to be 70–80 nm, their width is 2 nm, and their thickness corresponds to three monolayers.^21^ Additionally, the separation between the NPLs is estimated to be 1.5 nm, which is consistent with the value estimated from the small-angle XRD measurements as discussed above. This implies that lowering the reaction temperature does not change the thickness of the NPLs, but it induces asymmetric growth in one direction.

To further evaluate our hypothesis, X-ray photoelectron spectroscopy (XPS) measurements were carried out on the samples CS-60-60 and CS-60-80, and the results are presented in ESI Fig. S22.† In Fig. S22a,† the XPS peaks confirm the presence of C, N, Cs, Pb, and Br on the surface of both samples. In Fig. S22b,† we present the high-resolution image of the Pb 4f core level spectra. Two peaks were observed at for the CS-60-60 samples at 142.28 and 137.38 eV, corresponding to Pb 4f_5/2_ and Pb 4f_7/2_, respectively.^43^ On the other hand, the Pb 4f peaks of the CS-60-80 samples are shifted to higher binding energy values of 142.58 eV and 137.68 eV for Pb 4f_5/2_ and Pb 4f_7/2_, respectively. These results imply an improvement of 0.3 eV in the binding energy of Pb. In the case of the pristine samples, due to the limited availability of Br, the octahedra contain more Br defects, and the binding energy of Pb is lower. When the PbBr_2_ is preheated to a higher temperature, the PbBr_6_^4−^ octahedra are formed with few Br defects, and hence, there is an improvement in the binding energy of Pb.^26^ Additionally, additional peaks are observed at 140.18 eV and 135.38 eV, corresponding to the metallic Pb states in the case of CS-60-80.^23^ When the NPLs are exposed to the ambient atmosphere, the ligands on the surface are highly volatile, hence, they will detach from the surface of the NPLs; consequently, Pb atoms are present on the surface. Alternatively, this might be due to damage from the electron beam during the measurement.^24^ In Fig. S22c,† we present the binding energies of the Br 3d and Cs 4d energy states of both samples. In the case of the CS-60-60 sample, the binding energy of the Br 3d state is about 67.2 eV, and that of the Cs 4d state is about 74.4 eV. The reduction in the binding energy compared to the bulk samples indicates that the most of the atoms are present on the surface of the NPLs due to the high surface-to-volume ratio.^53^ Interestingly, in the case of the CS-60-80 samples, no peak corresponding to the Cs atoms is present on the surface of the NPLs. This might be due to some of the Cs ions being replaced by the oleylammonium bromide ions during the reaction.^53^

**Fig. 4:**
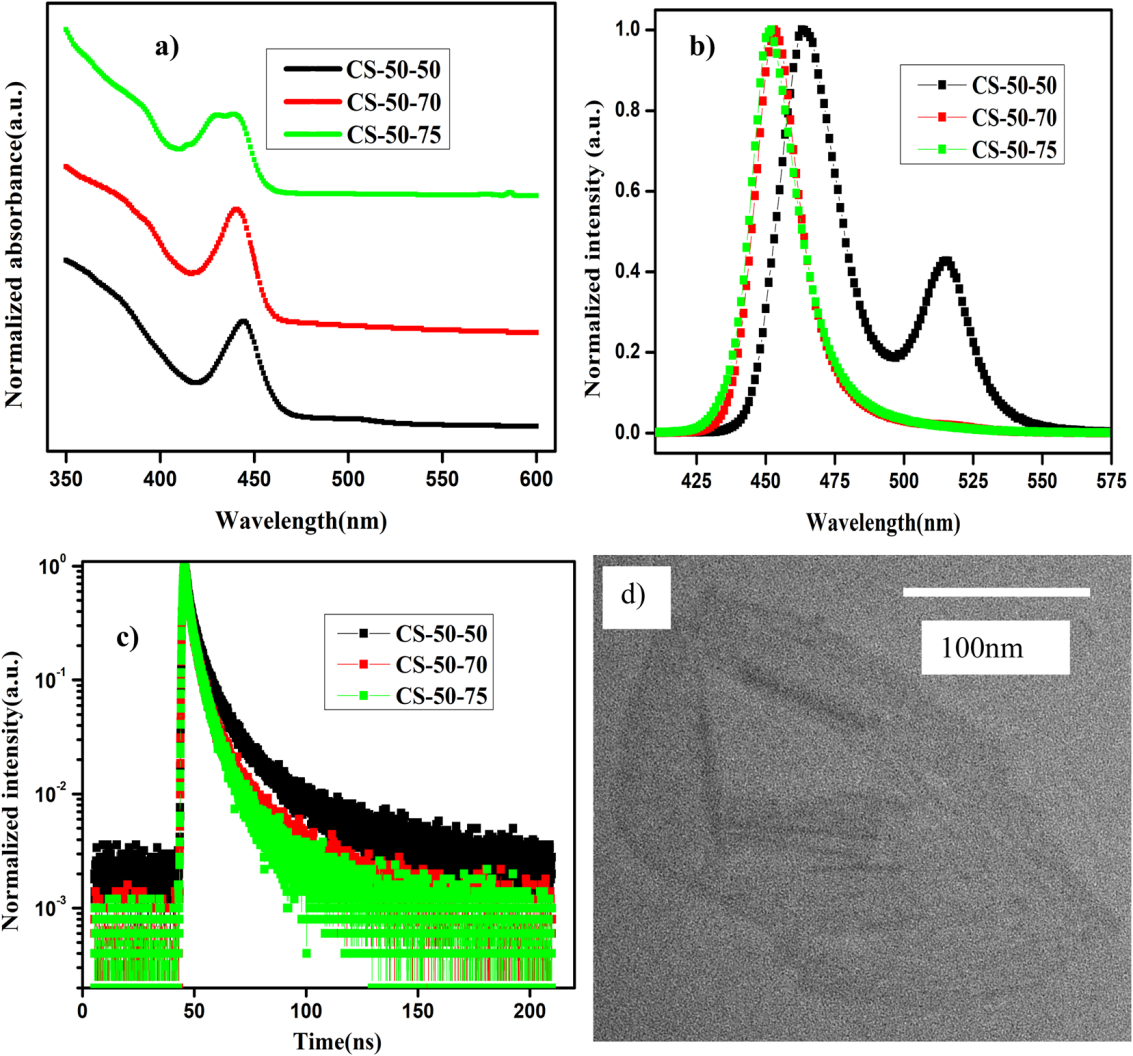
(a) UV-visible absorption and (b) normalized PL emission spectra of NPLs that are synthesized with different preheating temperatures for the PbBr_2_ precursor. (c) TRPL decay curves for the NPLs synthesized at 50 °C under different conditions. (d) TEM image of the sample CS-50-70.

In Fig. 4a and b, we present the absorption and normalized PL emission spectra of the NPLs that were synthesized at 50 °C with different PbBr_2_ precursor temperatures. In the case of the pristine samples (CS-50-50), the absorption spectrum has kinks at 510 nm and 460 nm (Fig. 4a, black curve), and the corresponding PL emission has peaks at 465 nm and 510 nm (Fig. 4b, black curve). Based on the knowledge obtained from the synthesis of the previous samples, we raised the PbBr_2_ precursor temperature 20 °C higher than the reaction temperature, and furthermore, we optimized the stirring time from 15 minutes to one hour. In this case, the absorption peak at 450 nm becomes more pronounced and the kink at 510 nm is suppressed (Fig. 4a, red curve); the corresponding PL peak appears at 450 nm, and the peak at 510 nm completely vanishes (Fig. 4b, red curve). The Stokes shift is reduced to 13 nm compared to 22 nm in the case of the pristine samples. Additionally, the FWHM is reduced to 20 nm, and PL intensity is appreciably improved. On the other hand, in the case of the CS-50-75 samples, the PL peak position is blue-shifted by 1 nm, but the PL peak intensity decreases appreciably (Fig. 4b, green curve). The main contributions to the upper valence band and conduction bands are the Pb 6p and Br 4p orbitals. Hence, the excess Pb and Br that are soluble at higher temperatures contribute to the additional states in the band spectrum. Thus, electron–hole recombination will take place from a higher energy level, which causes a blue shift in the PL emission. Additionally, the excess Br might cause additional trap states within the bandgap, which will improve the recombination of the charge carriers. In ESI Fig. S23,† we present photographic images under white light and UV light of the samples synthesized at 50 °C that were prepared with different PbBr_2_ preheating temperatures. Similarly, in ESI Fig. S24,† we present photographic images under white light and UV light of the same samples after being kept in the air for one month.

In Table 4, we present the PLQY values of the samples that were synthesized at 50 °C, which were prepared using PbBr_2_ precursor preheated to different higher temperatures. The PLQY of the as-synthesized CS-50-50 samples is about 40%. However, the as-synthesized samples consist of large-sized NCs; hence, the PLQY value is high. Over time, these large-sized NCs settled at the bottom of the vial, and the PLQY value of the NPLs dropped to 10%. To understand the origin of the poor PLQY, one must consider that the 2D materials will have a high surface-to-volume ratio, as explained above. This could render them susceptible to having more surface defects. Additionally, the reduced thickness of the NPLs also masks the dielectric screening, which evidenced by the large exciton binding energy.^21^ This further increases the nonradiative recombination as well as the scattering rates of the excitons with defects and phonons. In the case of the CS-50-70 samples, the PLQY value of the as-synthesized samples is 80%; this initial value is retained for two weeks, and then drops to 57% after a month. The PL intensities at an excitation wavelength of 400 nm for different values of absorption for the sample CS-50-70 are presented in ESI Fig. S25a,† and the integrated PL intensity as a function of absorbance for CS-50-70 and the reference sample coumarin 153 are presented in ESI Fig. S25b.† The enhancement in the PLQY value of the NPLs that were synthesized at 50 °C and 60 °C is greater than that of the NPLs that were synthesized at 70 °C and 80 °C, since the NPLs that were synthesized at 50 °C and 60 °C have relatively more atoms on the surface. These undercoordinated atoms on the surface lead to more surface defects. The PLQY values of the as-synthesized NPLs that were synthesized at 50 °C and 60 °C are very low. The solubility of PbBr_2_ is lower at lower temperatures, and the chemical unavailability of Br leads to formation of more defects. Since the defect density is higher due to the large surface-to-volume ratio in these samples, the excess Br that is soluble at higher temperatures will help to relieve the defects. In the case of the CS-50-75 samples, the PLQY is about 50% and maintains stability for one month. This indicates that in the case of CS-50-75, the excess Br atoms passivate the surface traps and improve the stability. In the case of the CS-50-70 samples, although the initial PLQY value is higher and maintains stability for two weeks, it drops to 57% after a month. The origin of the drop in the PLQY value is the exceptionally high surface-to-volume ratio of the NPLs. The atoms on the surface of the NPLs are protected by the ligands, which can easily detach from them upon being exposed to moisture.^38,53,54^ When the ligand is lost from the surface of the NPL, the atoms on the surface are more liable to detach from it.^51,52,55^ Since there are more atoms on the surface, the defect density will be larger; hence, the drop in the PLQY will be greater. Therefore, the availability of Br during the reaction is crucial for the formation of NPLs with fewer defects and to achieve a high PLQY. To analyze the effect of these band tail states, we calculated the value of trap density using eqn (1). The *E*_U_ value is estimated by fitting the exponential part for a logarithmic scale *α vs. E* graph. The *E*_U_ value of the pristine samples is 60 meV, which is larger compared to that of the CS-50-70 samples (47 meV). These results further demonstrate that the availability of Br helps to form defect-free NPLs, which consequently improve the PL properties.

**Table 4:** PLQY values of the samples that were synthesized at 50 °C with preheating of the PbBr_2_ precursor to different higher temperatures

Sample name	As-synthesized	1 week	2 weeks	1 month
CS-50-50	40.27	11.70	10.20	16.71
CS-50-70	89.69	73.57	85.78	56.98
CS-50-75	47.00	37.24	50.81	52.72

To further demonstrate the improvement in the PL properties, TRPL measurements were carried out, and the decay curves were well fitted using the bi-exponential decay function in eqn (2). The PL lifetimes of the short-lived and long-lived components and the distribution coefficients are presented in Table S4.† The PL decay of the pristine samples is relatively shorter and leads to an average lifetime of 3 ns, while the average lifetime of the CS-50-70 sample is 3.5 ns. Similarly, in the case of the CS-50-75 samples, the average lifetime of the charge carriers drops to 2.46 ns. This implies that the addition of excess Pb and Br might cause additional trap states; hence, the recombination of the charge carriers might prevail, and thus, the charge carrier lifetime will be shorter.

In ESI Fig. S26a–c,† we present the XRD analysis that was carried out on the samples prepared at a temperature of 50 °C. In ESI Fig. S26a,† we present the XRD pattern of the sample CS-50-50, which consists of only a high intensity single peak, indicating the high crystallinity of the synthesized NPLs. The peak was also shifted toward lower 2*θ* angle (28.6°) compared to that of cubic CsPbBr_3_ (30.2°). Using the most intense diffraction peak of the (200) plane, a unit cell size of 0.624 nm is estimated. The estimated size of the unit cell in NPL is larger by 0.024 nm than that of the bulk NC (0.6 nm). The elongation in the unit cell size is due to the passivation of the top and bottom facets of the unit cell by the oleylamine ligands.^53^ In addition, the low-intensity peak is observed at a 2*θ* angle of 6.5°. Hence, the separation between the NPLs is about 1.5 nm, which is similar to the estimated value in the case of CS-60-80. However, the intensity of the peak is lower compared to that of CS-60-80. Hence, we can infer that some of the NPLs form lamellar structures, and some of them form nanoribbons. Additionally, in the case of the CS-50-70 and CS-50-75 samples, an additional peak at 25.4° is observed, which corresponds to the formation of the Cs-rich compound Cs_4_PbBr_6_. It is most likely that some of the ligands are removed from the surface of the NPLs. These additional Cs ions can react with the Br ions and form the intermediate product Cs_4_PbBr_6_, which is non-emissive.

The morphology of the CS-50-70 sample was characterized using TEM, and it is presented in Fig. 4d. From this image, we can observe that the NPLs exhibit a ribbon-like morphology in addition to the lamellar structures of the NPLs that are synthesized at 60 °C. The lateral size of the nanoribbons is extended up to 200 nm. Additionally, these nanoribbons also exhibit curling and folding in some regions, which indicate their ultrathin nature.^5^ These nanoribbons are highly susceptible to the incident electron beam.

**Fig. 5:**
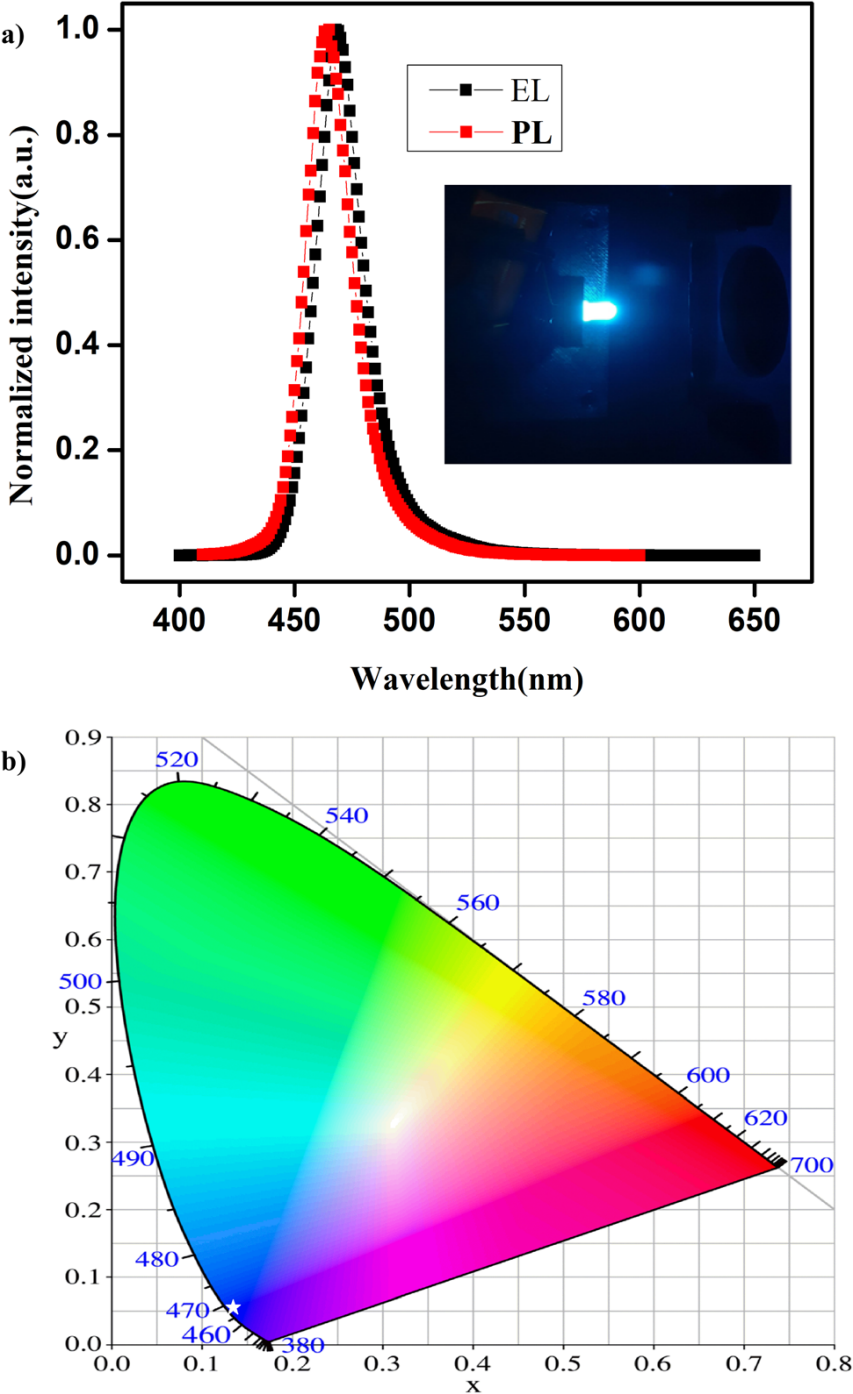
Comparison of the EL spectra of the thin-film and the PL spectra of the NPL dispersion in hexane (top). Inset shows a photographic image of the fabricated LED. (b) CIE coordinates for the EL spectrum.

For high-performance optoelectronic devices, a high-quality film is essential, and the PL properties should not deviate from the dispersion to the thin film.^56,57^ Taking advantage of their excellent optical properties and good stability, we have fabricated a down-conversion LED device using these NPLs that emit at 463 nm, and the typical normalized EL spectrum is presented in Fig. 5a. The inset shows a photographic image of an NPL-based LED in operation. The EL spectrum shows pure blue emission with a FWHM of 20 nm, indicating the effective exciton generation and recombination in the NPLs. Compared to that of the NPLs in the dispersion in hexane, the emission spectrum of the thin film shows a red shift of 4 nm in the LED device. However, we did not observe any additional peaks in the EL spectrum due to ripening or aggregation of the NPLs into large-sized NCs. In Fig. 5b, we present the Commission Internationale de l'Eclairage (CIE) chromaticity diagram of the device. The white star represents the CIE colour coordinate of the LED emission, and the coordinates are (0.135, 0.045), which is close to the NTSC standard for blue light (0.14, 0.08).

## 4. Conclusions

In summary, we have synthesized NPLs using the VALT method over the temperature range of 50 to 80 °C. These NPLs were synthesized by preheating the PbBr_2_ precursor to a higher temperature than the reaction temperature. The high solubility of Br at high temperature results in the formation of PbBr_6_^4−^ octahedra with fewer defects, as well as changing the growth dynamics. Consequently, the synthesized NPLs exhibit a wavelength emission range of 450–475 nm with an FWHM of 10 nm. The PLQY of the synthesized NPLs is about 80% at 450 nm and reaches near unity at a wavelength of 485 nm. Additionally, these NPLs exhibit excellent stability for a period of one month. We also fabricated down-conversion LEDs, whose EL peak shows only a slight shift from the PL peak of the dispersion. We believe that this work highlights the important role of the availability of Br during the reaction for the formation of defect-free NPLs to achieve high PLQY and stability.

## Conflicts of interest

The authors declare that they have no conflict of interest.

## Supplementary Material

NA-004-D2NA00354F-s001
