# Supplementary material for: In situ synthesis of high-quantum-efficiency and stable bromide-based blue-emitting perovskite nanoplatelets
Source: Nanoscale Adv. 2022 Oct 13;4(22):4766–81. doi: 10.1039/d2na00354f (PMC9642352; doi:10.1039/d2na00354f)
Supplement: NA-004-D2NA00354F-s001 [file NA-004-D2NA00354F-s001.pdf]

Electronic Supplementary Material (ESI) for Nanoscale Advances.
This journal is © The Royal Society of Chemistry 2022

Supporting Information for

In situ synthesis of high quantum efficiency and stable bromide-based blue-emitting perovskite nanoplatelets

Srinivasa Rao Pathipati[1], Muhammad Naeem Shah[2], Syed Akhil[3] and Nimai Mishra[3]

[1]Laboratory for Semiconductor Research, Department of Physics, School of Applied Science and Humanities, Vignan's Foundation for Science, Technology, and Research (Deemed University), Vadlamudi, Guntur, Andhra Pradesh, India-522213.

[2]College of Electronics and Information Engineering, Shenzhen University, Shenzhen, Guangdong, P. R. China -518000.

[3]Department of Chemistry, SRM University-AP, Andhra Pradesh, Neerukonda, Guntur (Dt), Andhra Pradesh 522240, India.

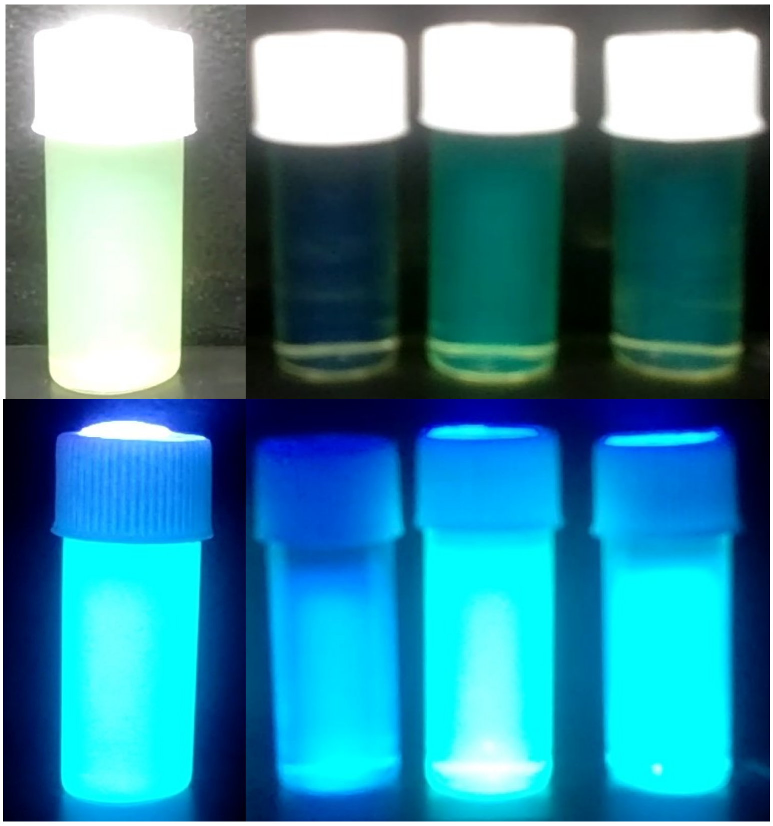

Figure S1: Photographic image of the as-synthesized samples CS-80-80, CS-80-87, CS-80-95, CS-80-100 under white light (top) and under UV light (bottom).

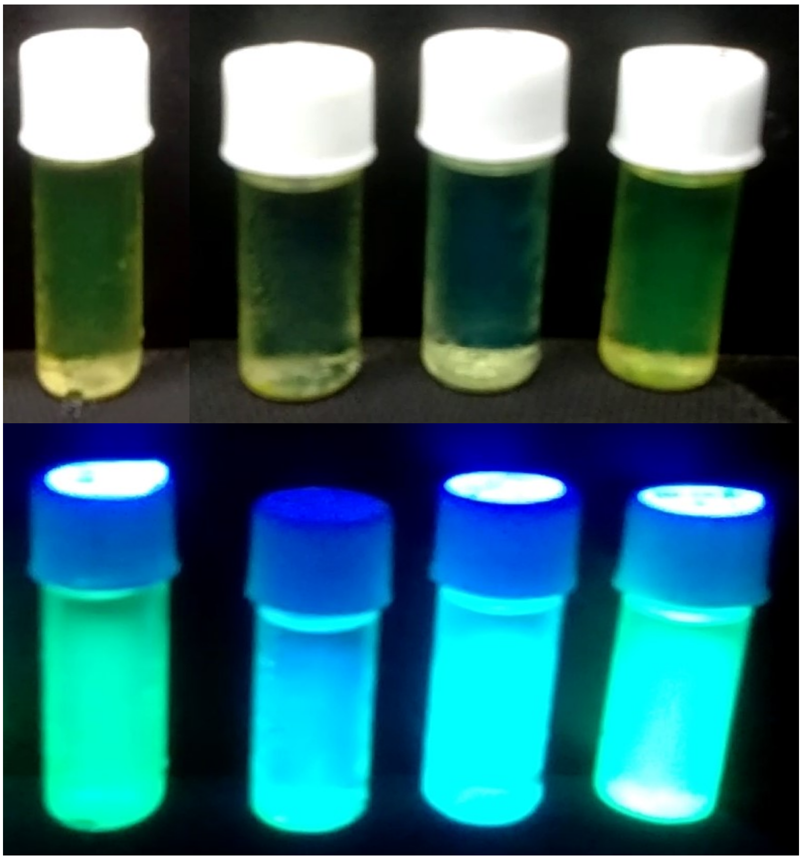

Figure S2: Photographic image of the samples CS-80-80, CS-80-87, CS-80-95, CS-80-100 which are kept in ambient atmosphere for a month under white light (top) and under UV light (bottom).

Table S1: The short-lived and long-lived PL lifetimes and the distribution coefficients of the samples which are synthesized at 80ºC under different conditions.

Sample	C_1	τ_1	C_2	τ_2	Average
CS-80-80	465.6229	6.59E-09	781065.7	3.25E-09	3.26E-09
CS-80-87	4.34E+07	2.48E-09	1591.093	5.83E-09	2.49E-09
CS-80-95	67904.33	4.00E-09	161.8602	7.52E-09	4.01E-09
CS-80-100	9.43E+06	2.80E-09	1605.605	6.10E-09	2.80E-09

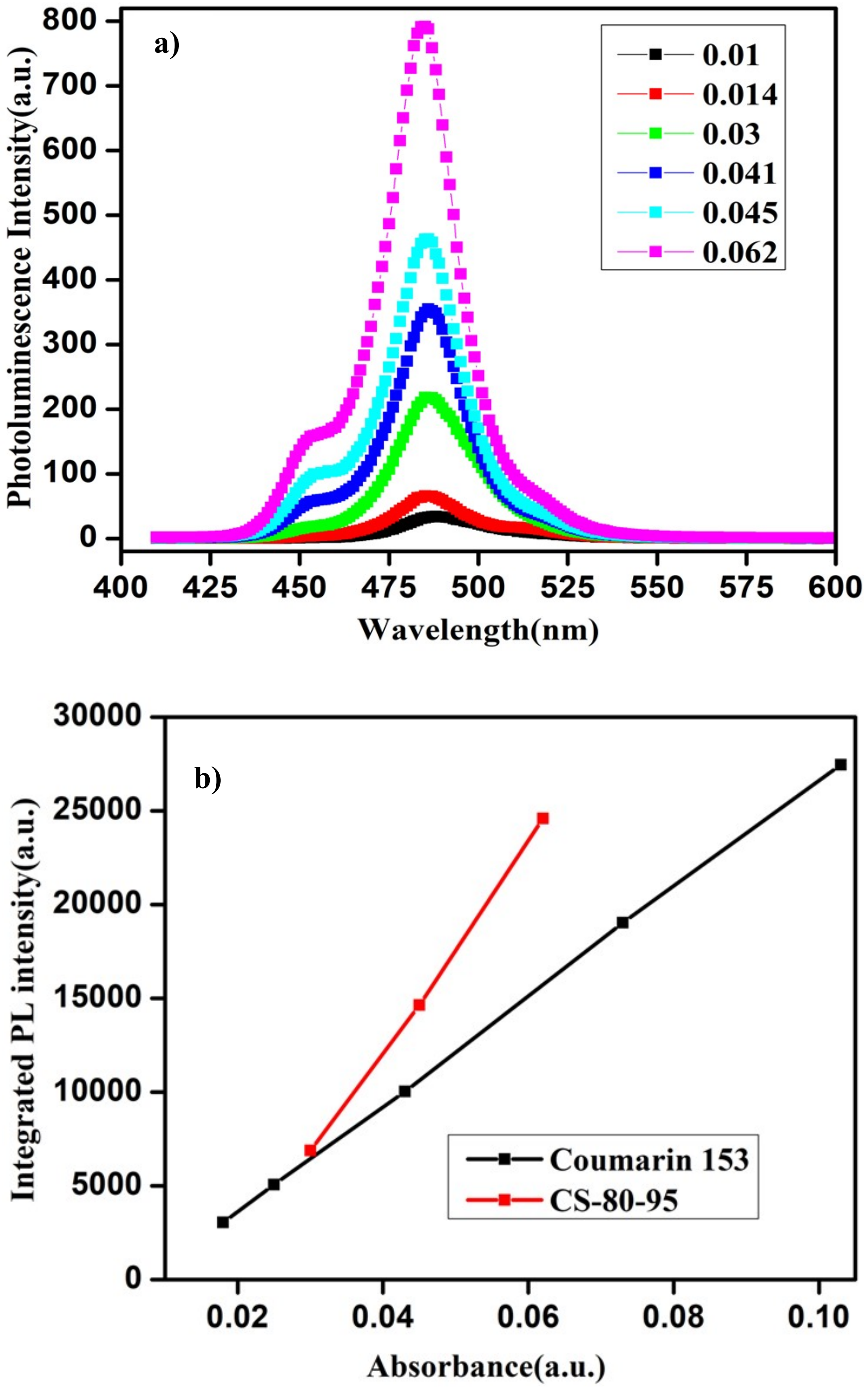

Figure S3: PL intensity as a function of the wavelength for different absorption at an excitation wavelength of 400nm for the sample CS-80-95 b) Integrated PL intensity vs absorbance for Coumarin 153 and CS-80-95 sample.

In Figure S3a), we present the PL vs wavelength for different absorbance values at an excitation wavelength of 400nm for the sample CS-80-95. It can be seen from this image that

the intensity of the PL peak increases with increasing the concentration of the NCs. Besides, the PL peak is blue-shifted with increasing the concentration of NPLs. At low concentrations of NPLs (low absorbance), few charge carriers are excited which will fill the trap states. Consequently, the emission is taking place from the midgap states; hence the low energy photons are emitted. At higher concentrations (high absorbance), a large number of charge carriers are excited which will fill first the midgap states, and the remaining charge carriers occupy the higher energy levels above the bandgap. Consequently, the photon emission is taking place from higher energy levels. Hence the PL emission peak is blue-shifted with increasing the concentration of the NPLs.

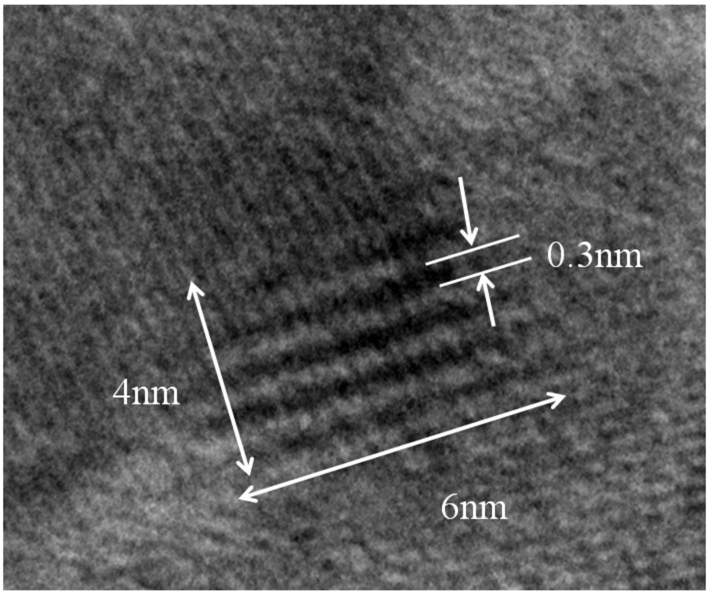

Figure S4: TEM image of the single NPL. The separation between the (200) atomic planes is estimated as 0.3nm.

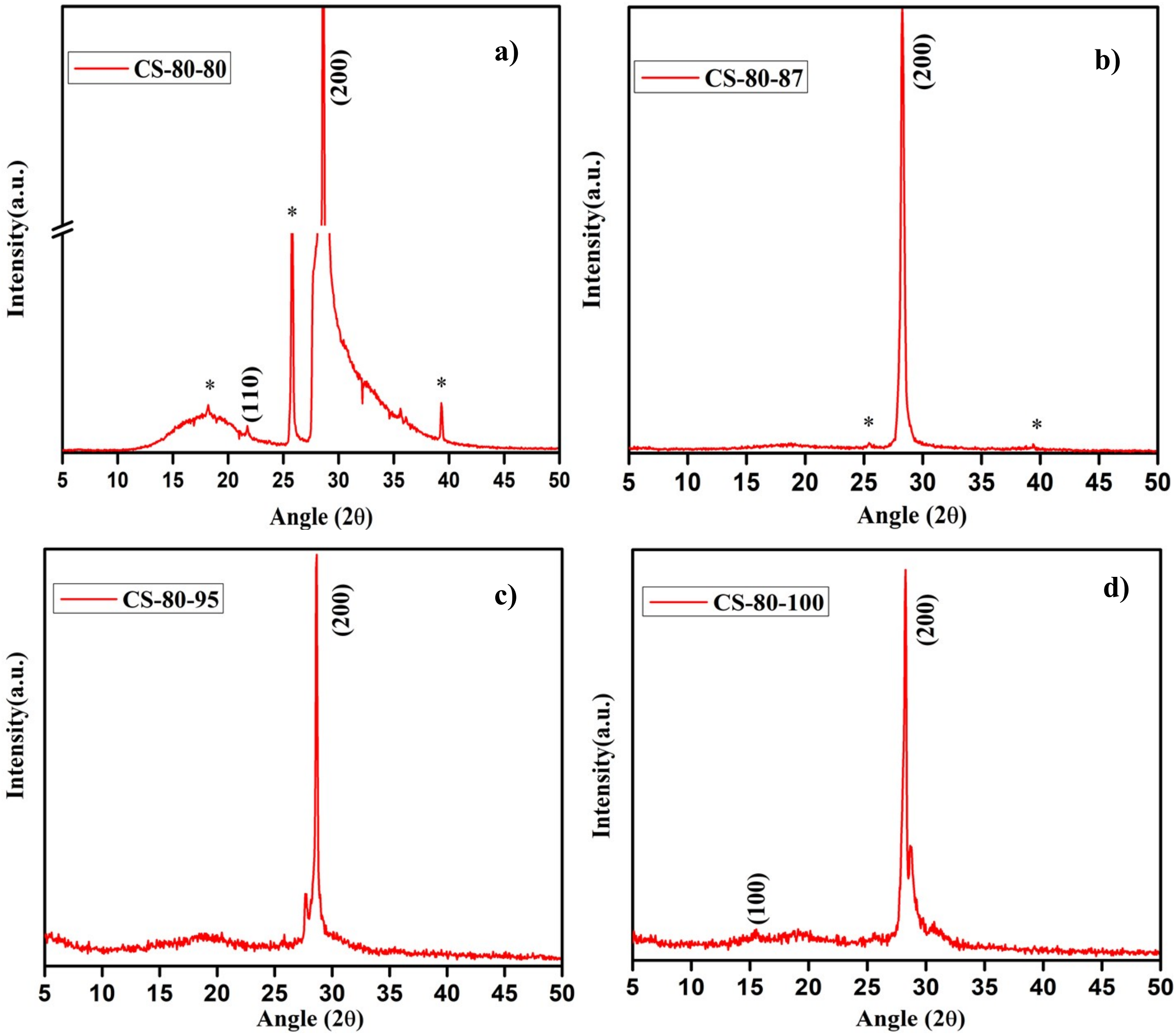

Figure S5: a)-d) XRD spectra of the NPLs which are synthesized at a temperature of 80ºC in which the $PbBr_2$ precursor is preheated to a different temperature.

In Figure S6a, we compare the FTIR spectra of the CS-80-80 and CS-80-95 samples and the FTIR spectra of all the samples are presented in Figure S6b. The peak that appears at 3078cm^{-1} can be attributed to the C-H stretching vibration mode of the Olefin. The presence of Olefin is due to the residual Octadecene solvent. The peak at 3002 cm^{-1} corresponds to the N-H stretching vibration mode of the amine group. The peaks that appeared at 2963cm^{-1}, 2921cm^{-1}, and 2852cm^{-1} correspond to the C-H stretching of the methylene. The peak that appeared at 1545cm^{-1} is due to the presence of the N-H scissoring, which confirms the presence of the Oleylamine on the surface of the NCs[1]. The peaks at 1459cm^{-1}, 1404cm^{-1}, and 1373cm^{-1} can be attributed to the carboxylate (COO^-) and methyl (CH_3) groups. The peak at 986cm^{-1} corresponds to the C-H vibration mode and the peak at 910cm^{-1} corresponds to the C-N and C=O stretching vibration that comes from the presence of Oleic acid and Oleylamine. The peak at 721cm^{-1} corresponds to the $–CH_2$ vibration of the alkyl chain[2]. It can be observed from the FTIR spectra, that the intensity of the peak in the wavenumber range from 1300-1600cm^{-1} increase for all samples relative to the pristine sample, which represents the functional groups that are present on the surface of the NCs, also increases. It implies with increasing the $PbBr_2$ precursor temperature during the synthesis increases and Br availability improves the atoms on the surface and hence the ligands that are attached to the atoms on the surface also increase. Hence the intensity of the peaks that represent the functional groups on the surface of the perovskite also improves.

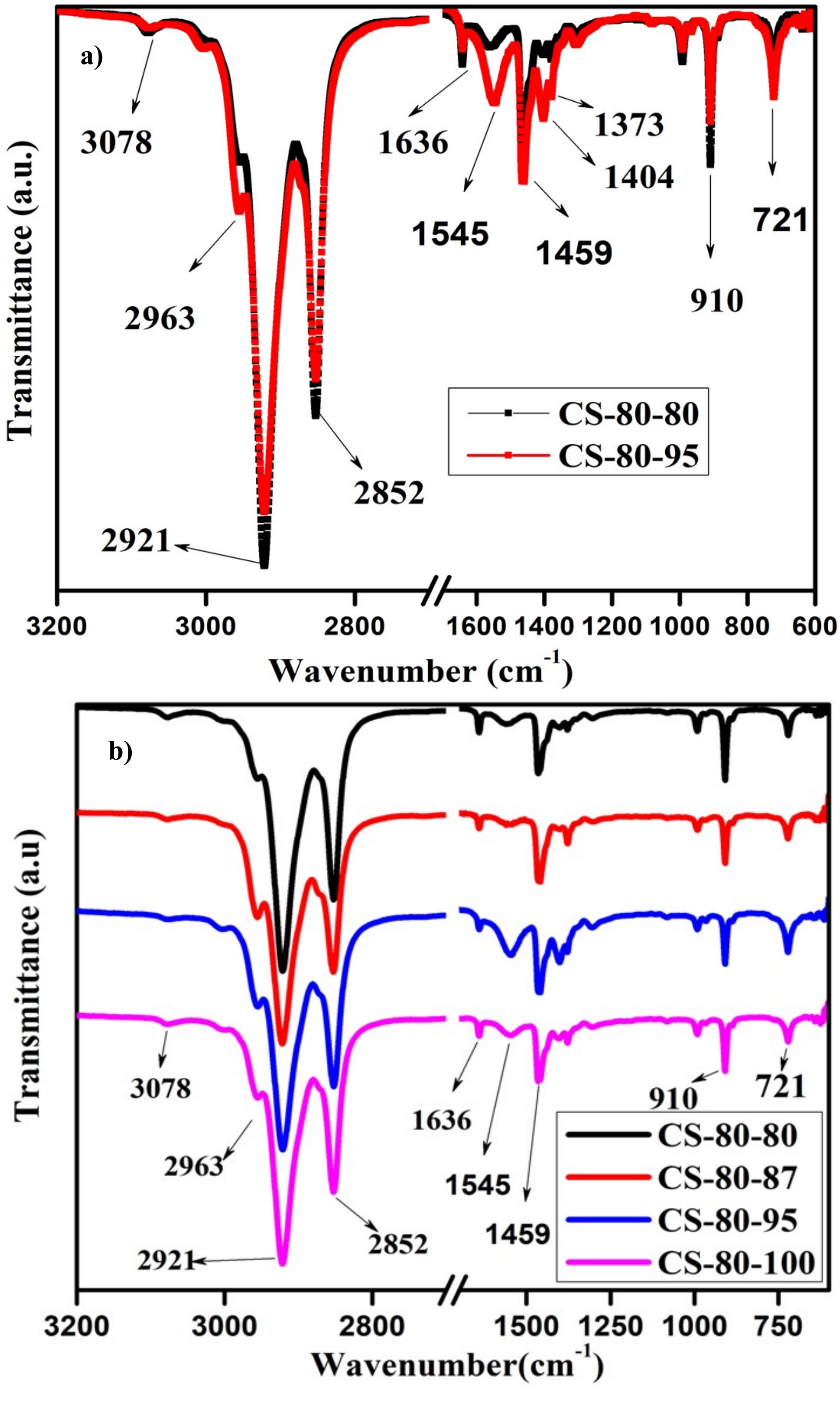

Figure S6: a) Comparison of the FTIR spectra of NPLs CS-80-80 and CS-80-95 b) FTIR spectra of NCs which are synthesized at 80ºC under different conditions.

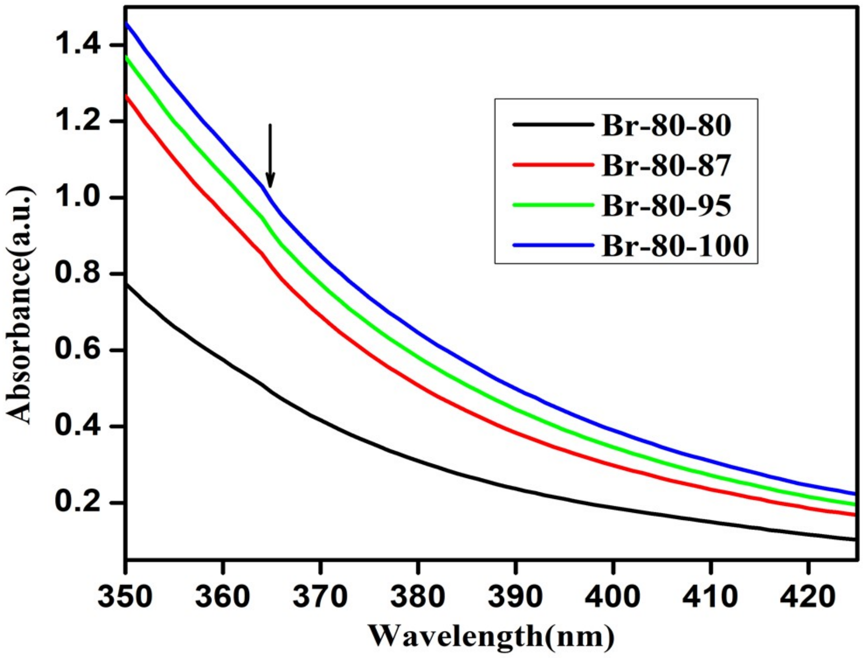

Figure S7: UV-Vis absorption spectrum of the $PbBr_2$ precursor solvent in Octadecene which is heated to a different temperature. The kink at 362nm shows the formation of the octahedra.

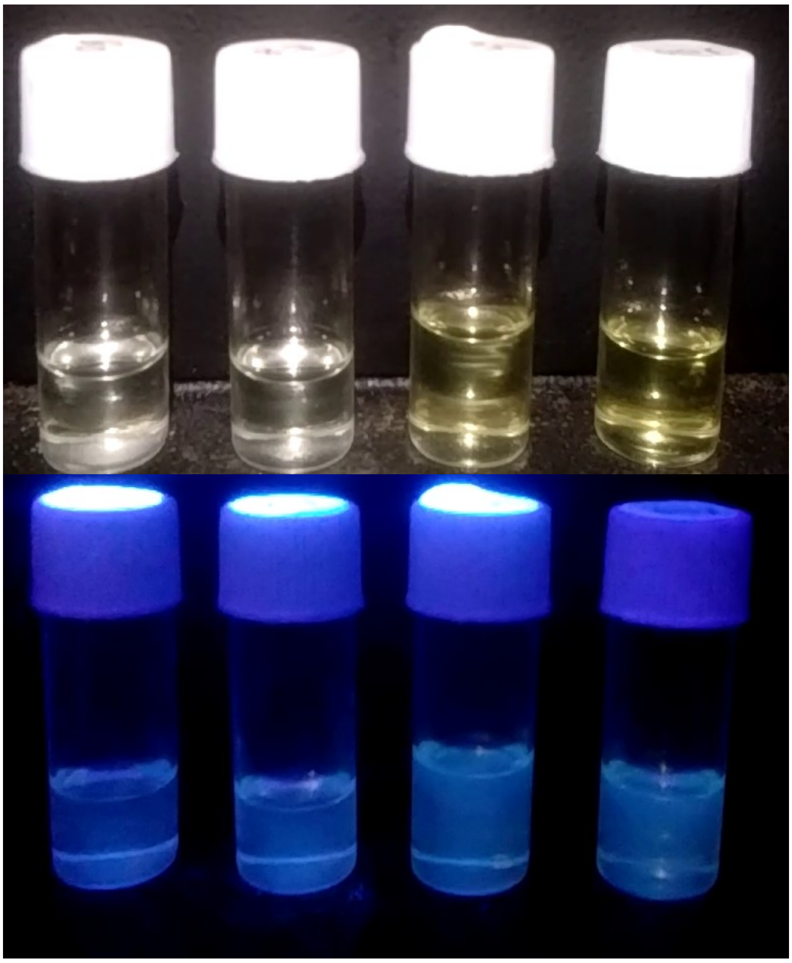

Figure S8: Photographic image of the $PbBr_2$ dissolved in Octadecene in the presence of ligands which is heated to a different temperature under white light (top) and under UV light (bottom). The $PbBr_2$ precursor temperatures are 80, 87, 95 and 100 (left to right).

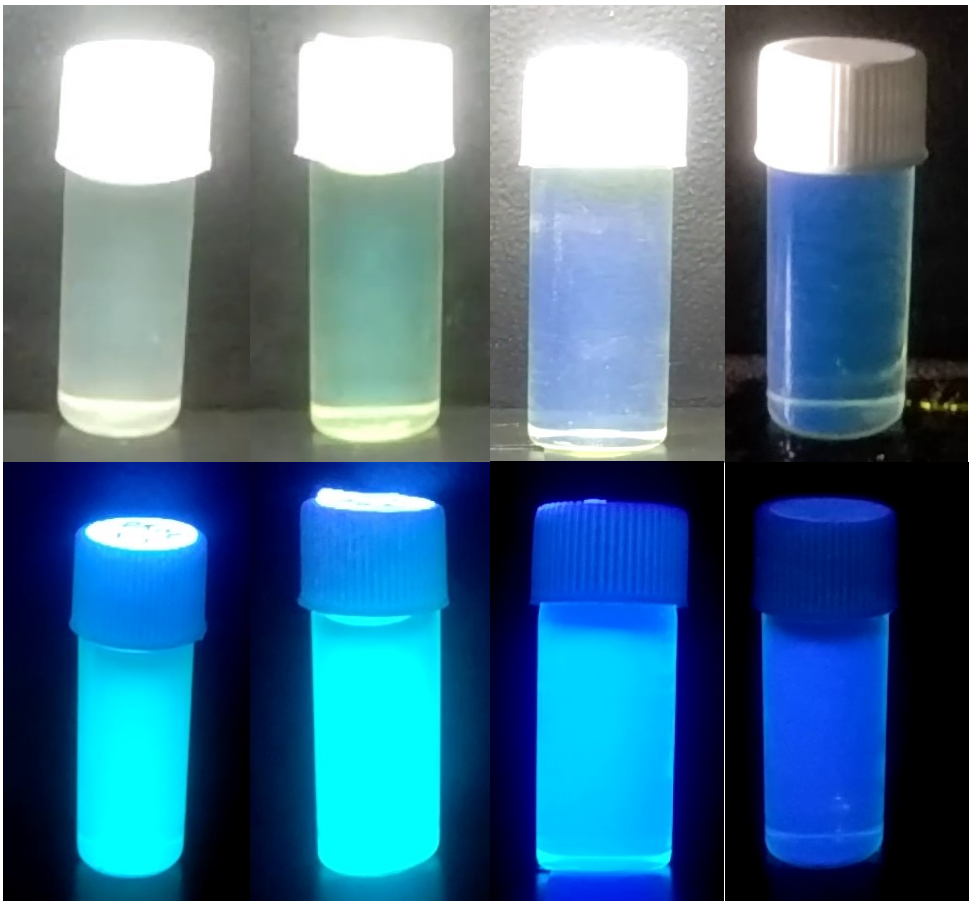

Figure S9: Photographic image of the as-synthesized samples CS-70-70, CS-70-80, CS-70-87, CS-70-90 under white light (top) and under UV light (bottom).

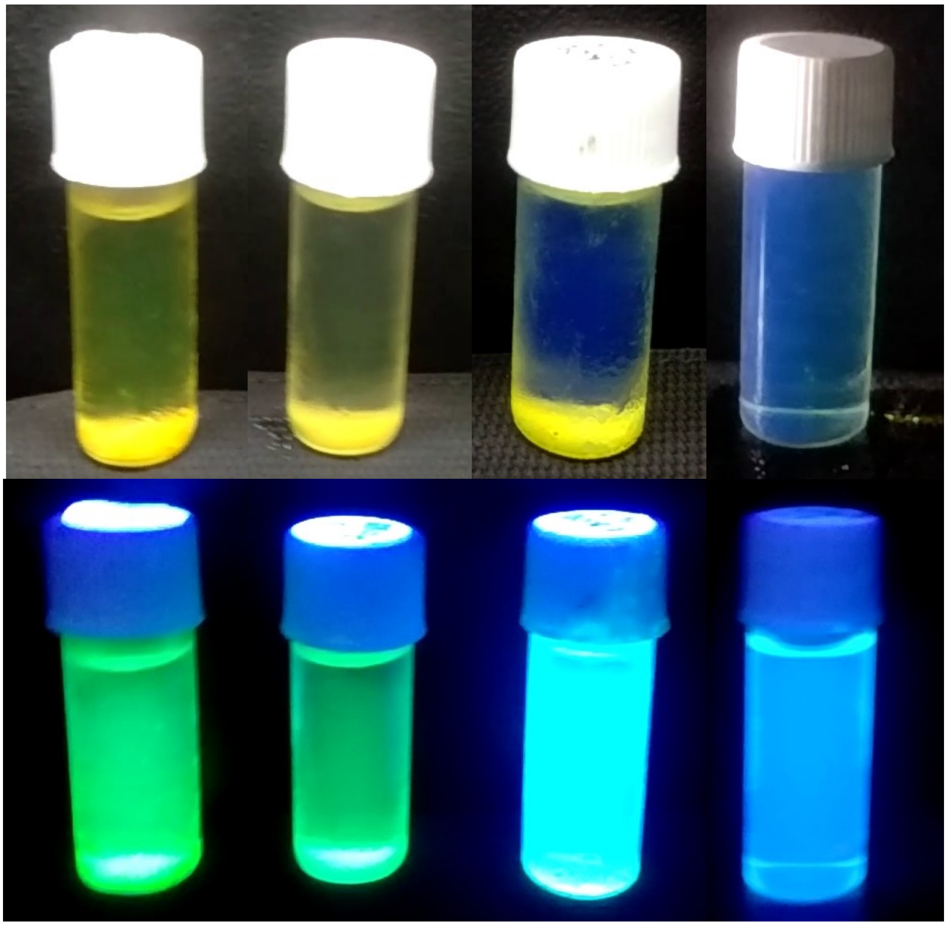

Figure S10: Photographic image of the samples CS-70-70, CS-70-80, CS-70-87, CS-70-90 which are kept in ambient conditions for a month under white light (top) and under UV light (bottom).

Table S2: The short-lived and long-lived PL lifetimes and the distribution coefficients samples which are synthesized at 70ºC under different conditions.

Sample	C_1	τ_1	C_2	τ_2	Average
CS-70-70	217.9884	7.30E-09	414402.2	3.57E-09	3.58E-09
CS-70-80	3.74E+07	2.55E-09	3214.734	5.68E-09	2.55E-09
CS-70-87	6.35328	1.15E-08	15778.32	4.67E-09	4.67E-09
CS-70-90	69.7954	9.52E-09	70.475	9.85E-09	9.69E-09

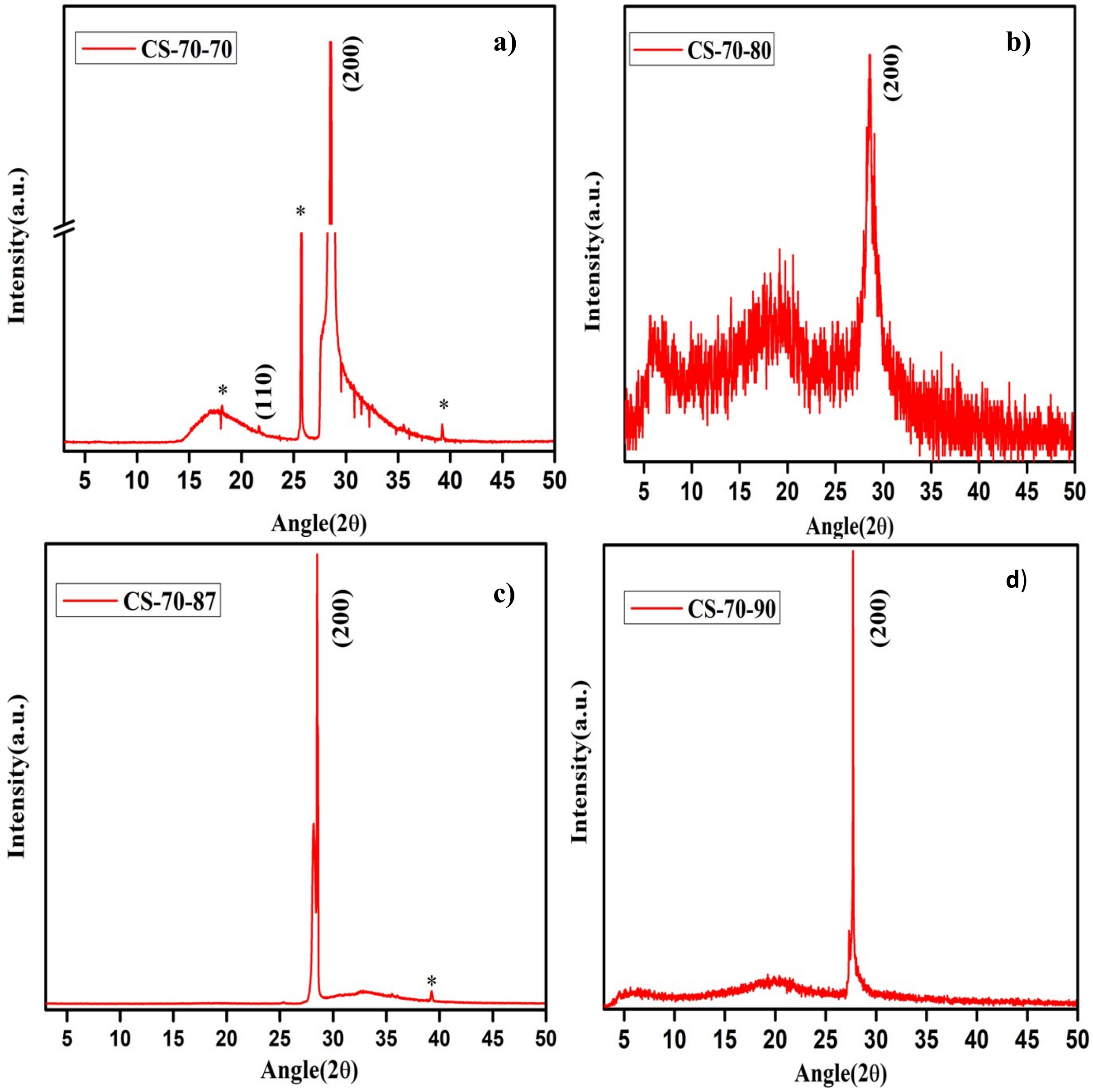

Figure S11: a)-d) XRD spectra of the NPLs synthesized at a temperature of 70ºC which are prepared by heating the $PbBr_2$ precursor temperature.

In Figure S12a, we compare the FTIR spectra of the samples CS-70-70 and CS-70-90 and FTIR spectra of all the samples which are synthesized at 70ºC are presented in SI Figure S12b. These NCs also exhibit the peaks in the FTIR spectra same as the NCs that are synthesized at 80ºC as explained above. In this case the intensity of the peaks increases in the wavenumber range from 1300-1600cm^{-1}, further confirms the presence of more ligands on

the surface. The presence of more number of ligands on the surface due to formation of the well coordinated $PbBr_6^{4-}$ octahedra.

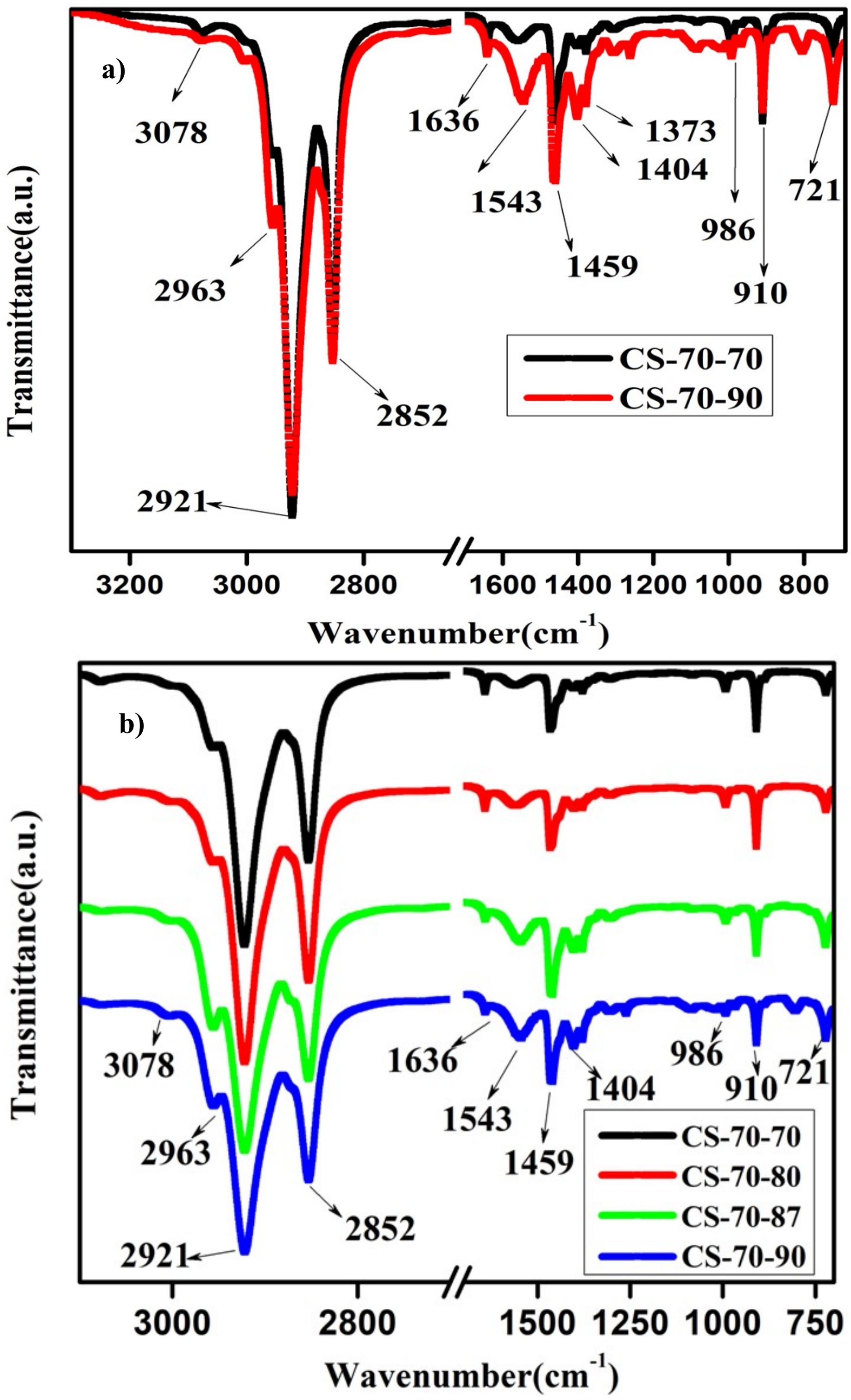

Figure S12: a) Comparison of the FTIR spectra of CS-70-70 and CS-70-90 and b) FTIR spectra of NCs synthesized at 70ºC prepared under different conditions.

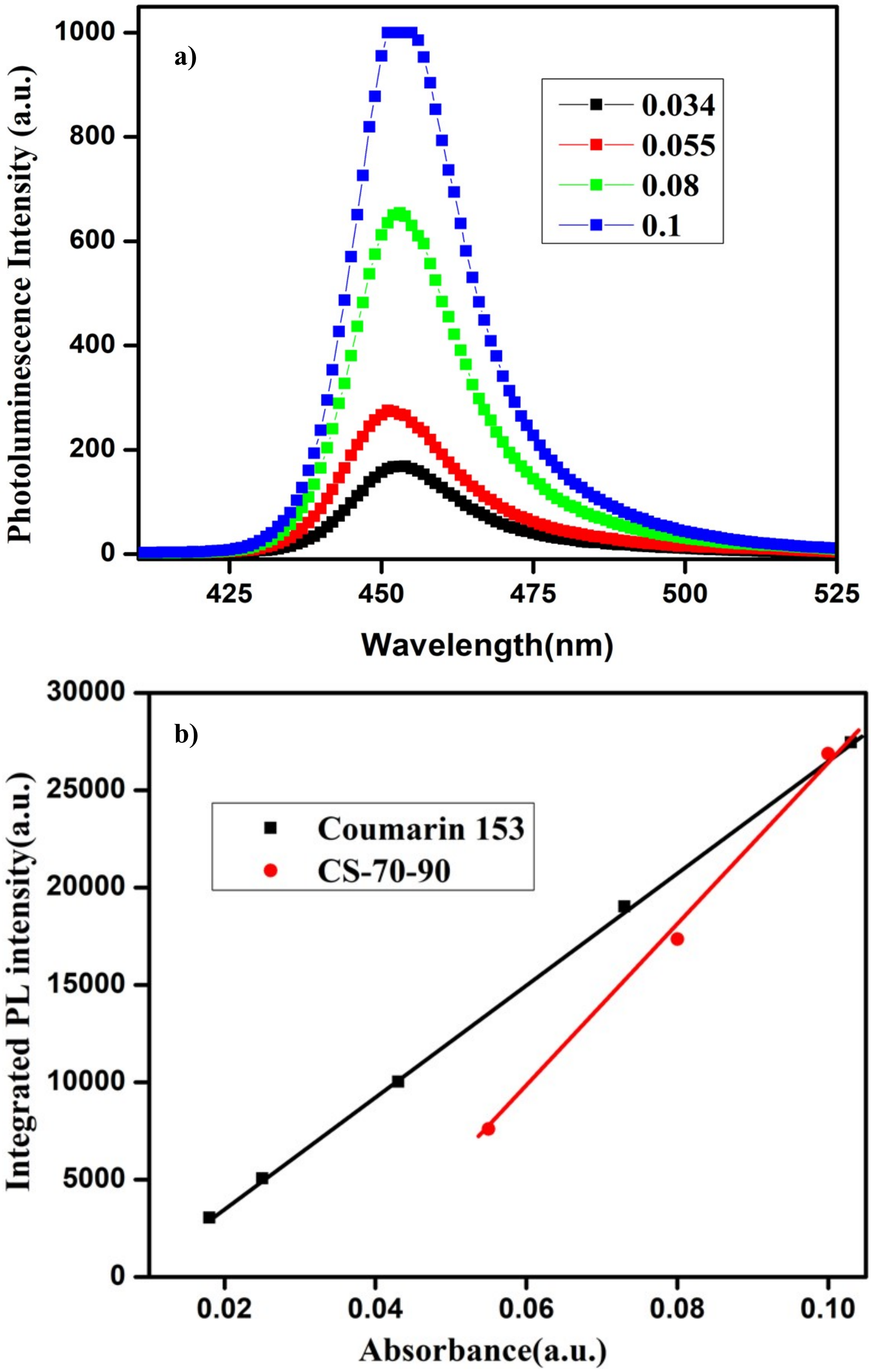

Figure S13: a) PL intensity as a function of wavelength for different absorbance at an excitation wavelength of 400nm b) Integrated PL intensity vs absorbance for Coumarin 153 and CS-70-90.

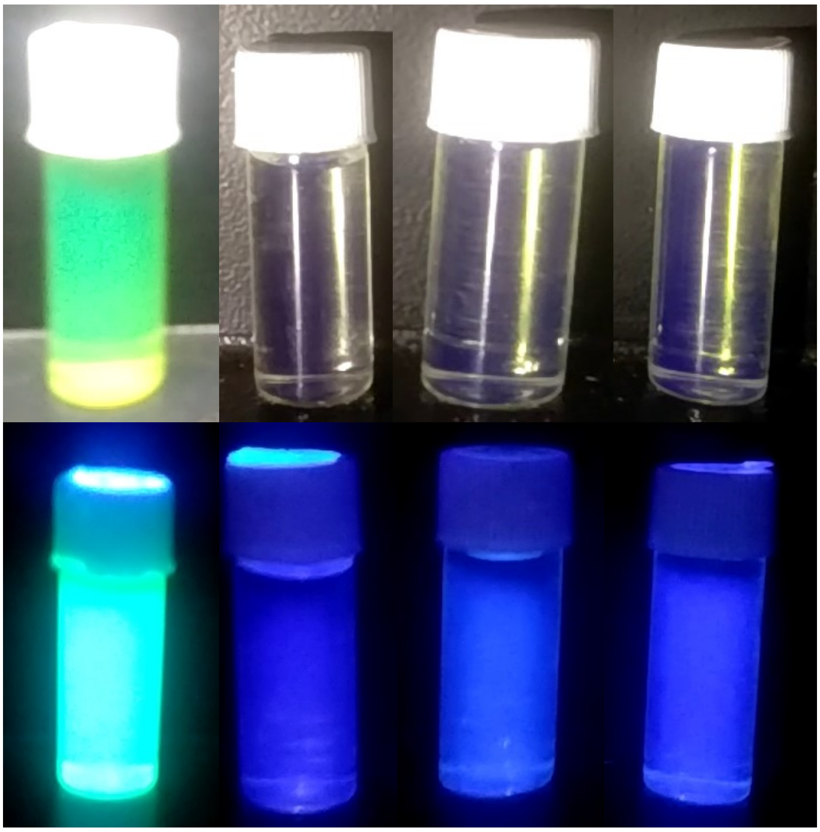

Figure S14: Photographic image of the as-synthesized samples CS-60-60, CS-60-70, CS-60-75, CS-60-80 under white light (top) and under UV light (bottom).

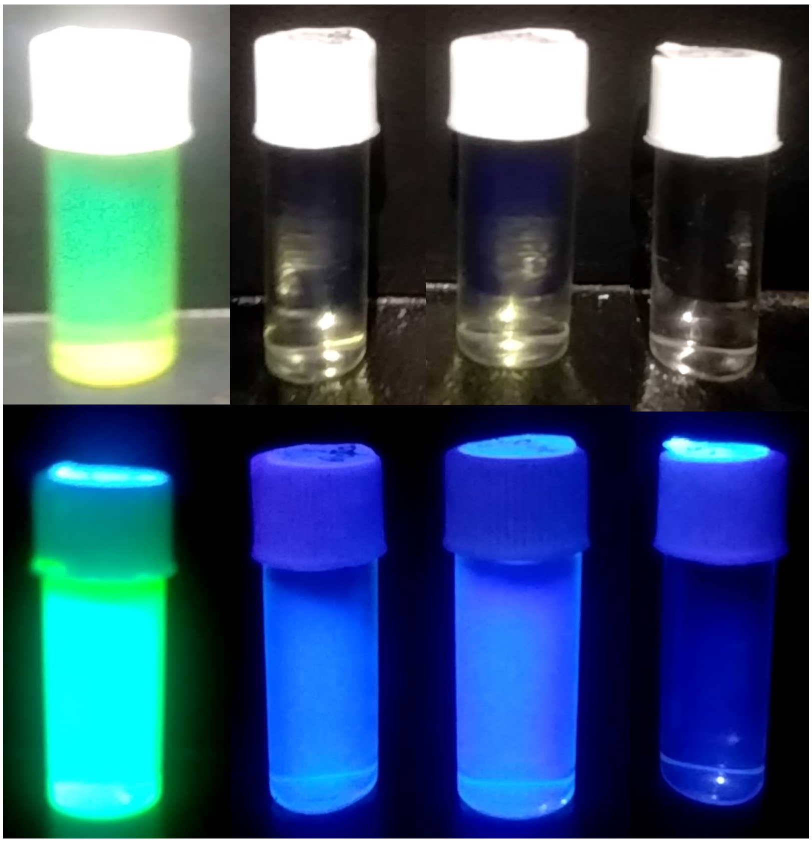

Figure S15: Photographic image of the samples CS-60-60, CS-60-70, CS-60-75, CS-60-80 which are kept in ambient conditions for a month under white light (top) and under UV light (bottom).

Table S3: The short-lived and long-lived PL lifetimes and the distribution coefficients of the samples which are synthesized at 60ºC under different conditions.

Sample	C_1	τ_1	C_2	τ_2	Average
CS-60-60	2.68	1.47E-08	9698	4.88E-09	4.89E-09
CS-60-70	2.01	1.36E-08	631053	3.34E-09	3.34E-09
CS-60-75	6.52	1.52E-08	15071	4.72E-09	4.73E-09
CS-60-80	12.2	9.93E-09	39035	4.08E-09	4.08E-09

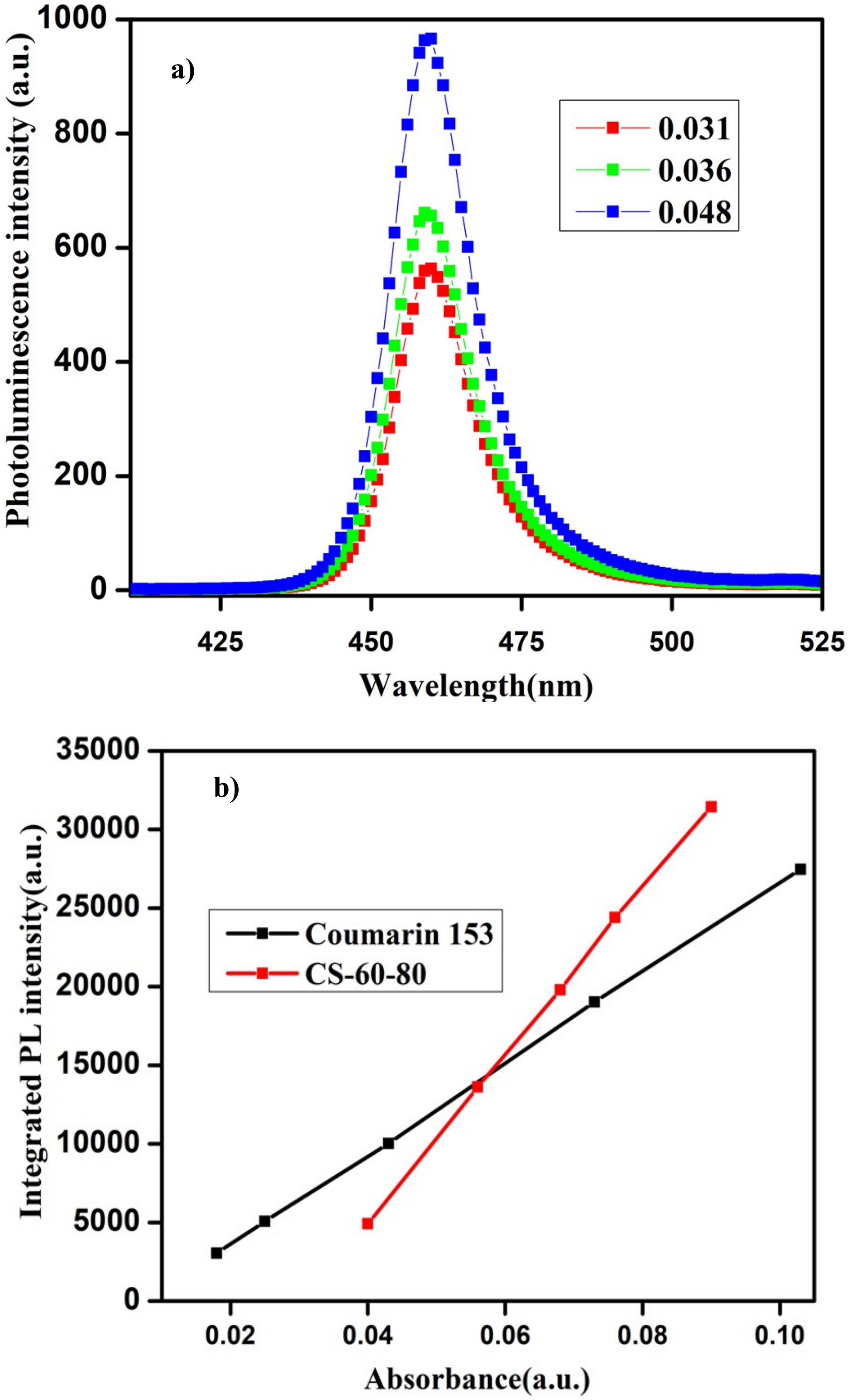

Figure S16: a) PL intensity vs wavelength for different values of absorption for the sample CS-60-80 b) Integrated PL intensity vs absorbance for Coumarin 153 and CS-60-80 samples.

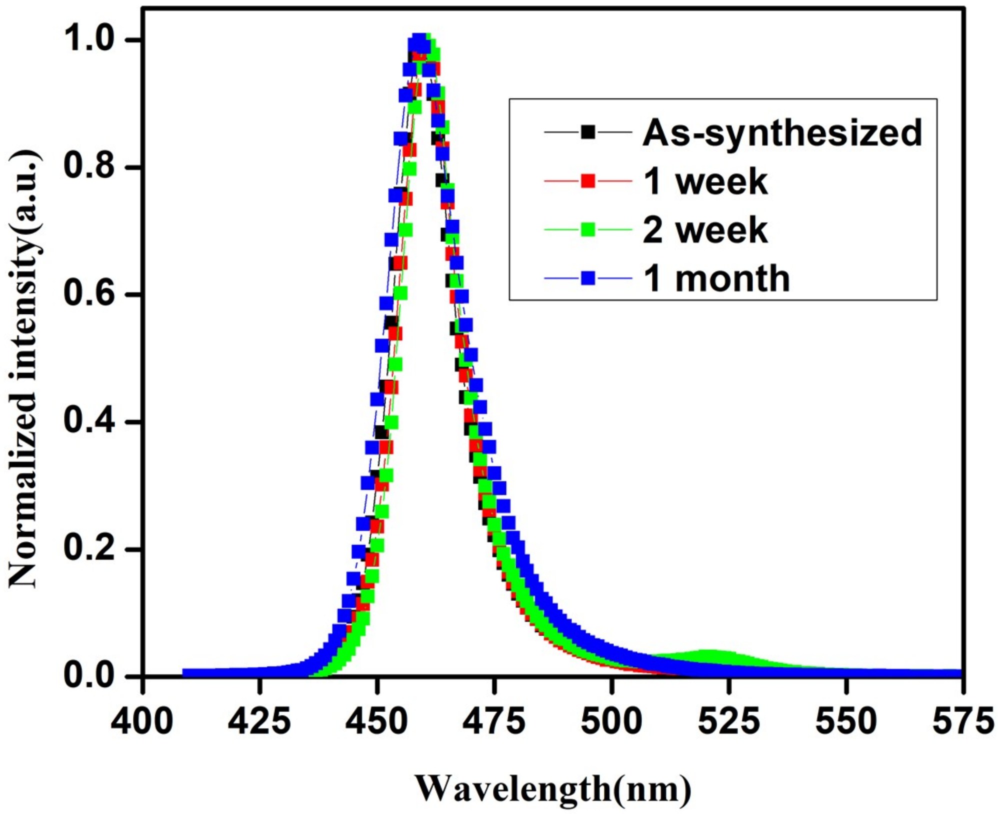

Figure S17: Photoluminescence vs wavelength for CS-60-80 samples over a period of time.

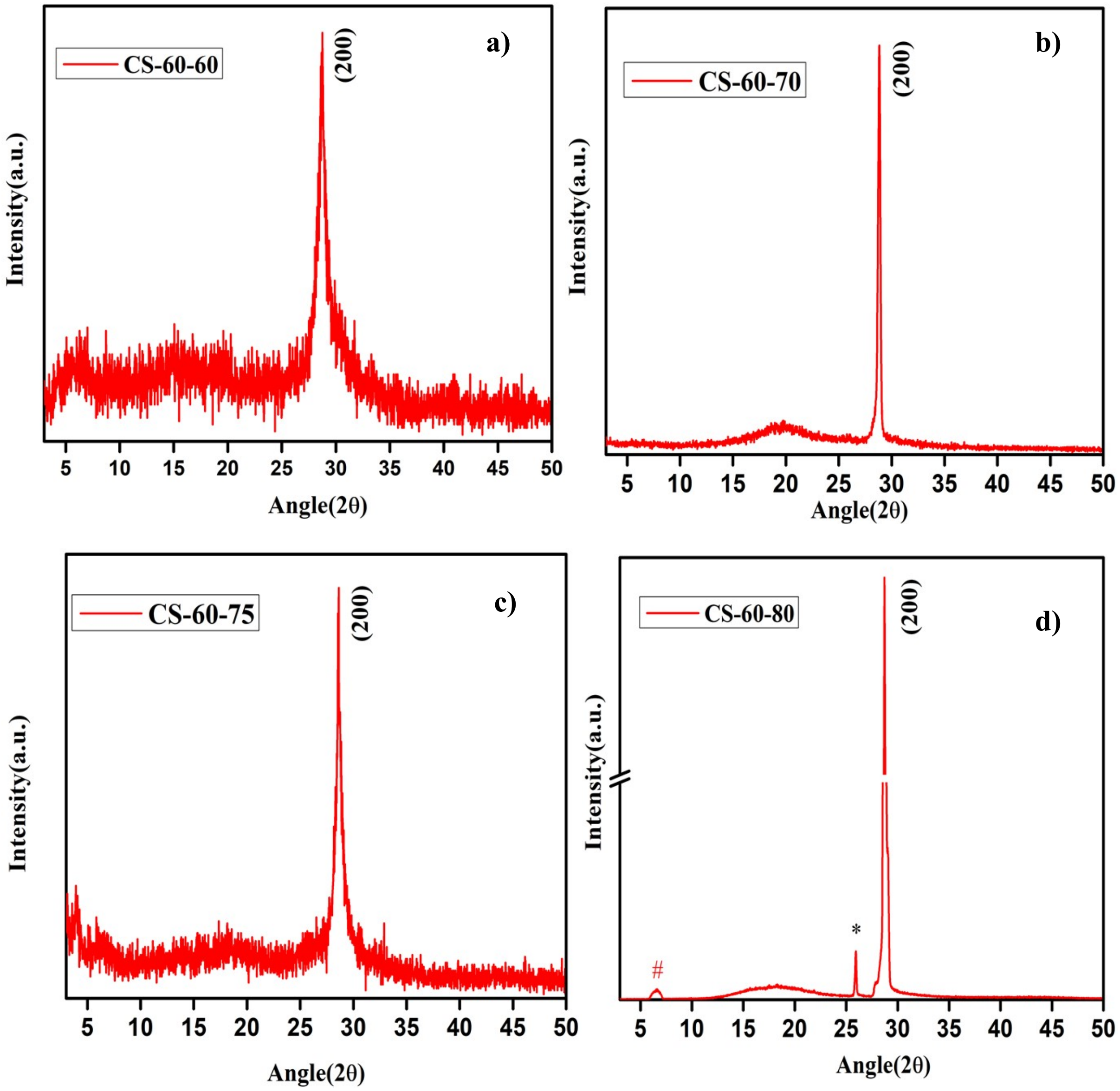

Figure S18: a)-d) XRD spectra of the NPLs which are synthesized at a temperature of 60ºC under different preheated temperatures of $PbBr_2$ precursor.

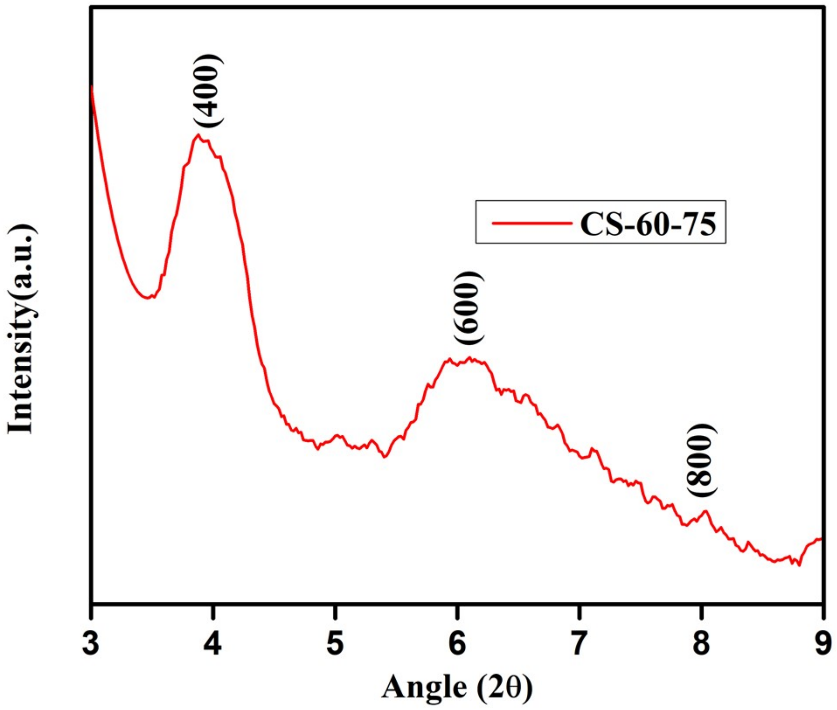

Figure S19: Small-Angle XRD pattern of CS-60-75 sample.

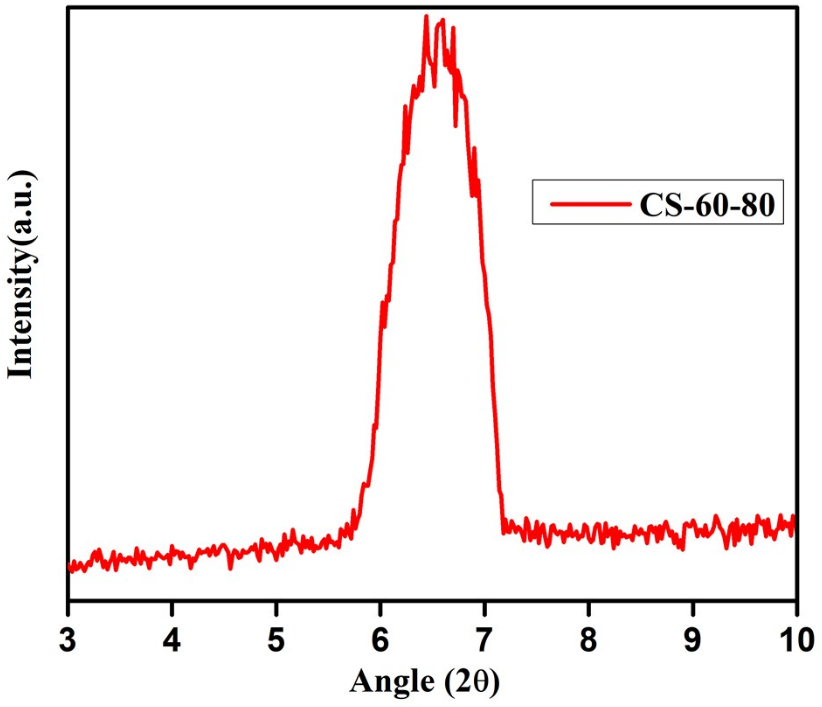

Figure S20: XRD Spectra of CS-60-80 deposited on silicon substrates at low angles.

In Figure S21a, we compare the FTIR spectra of the pristine samples (CS-60-60) and CS-60-80 samples and FTIR spectra for all samples that are synthesized at 60ºC are presented in Figure S21b. The peaks in the FTIR spectra for these samples appear at the same number as for the samples which are synthesized at 70ºC and 80ºC as discussed above. From the FTIR spectra, it is evident that the intensity of the peaks has increased substantially over all wavenumber range, when the $PbBr_2$ precursor temperature is raised to a higher value. These results confirm the presence of more ligands on the surface and hence more functionalities on the surface. These result further support the phenomenon that increasing the $PbBr_2$ helps to form the defect-free NPLs and presence of more ligands on the surface. The presence of functional groups improves the intensity of the peaks in the FTIR spectrum.

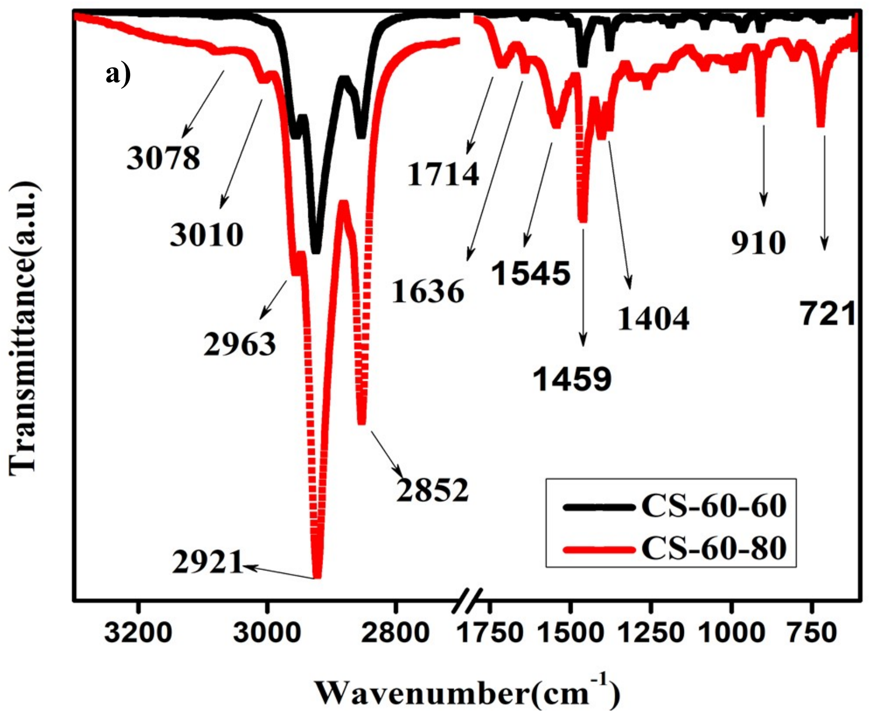

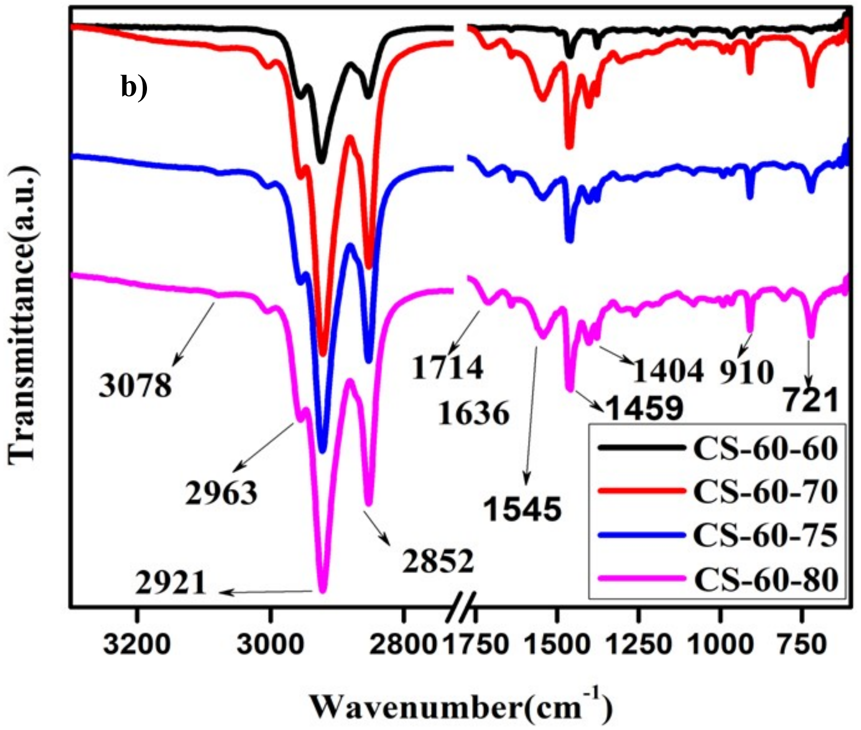

Figure S21: FTIR Spectra of the NPLs synthesized at 60ºC under different conditions.

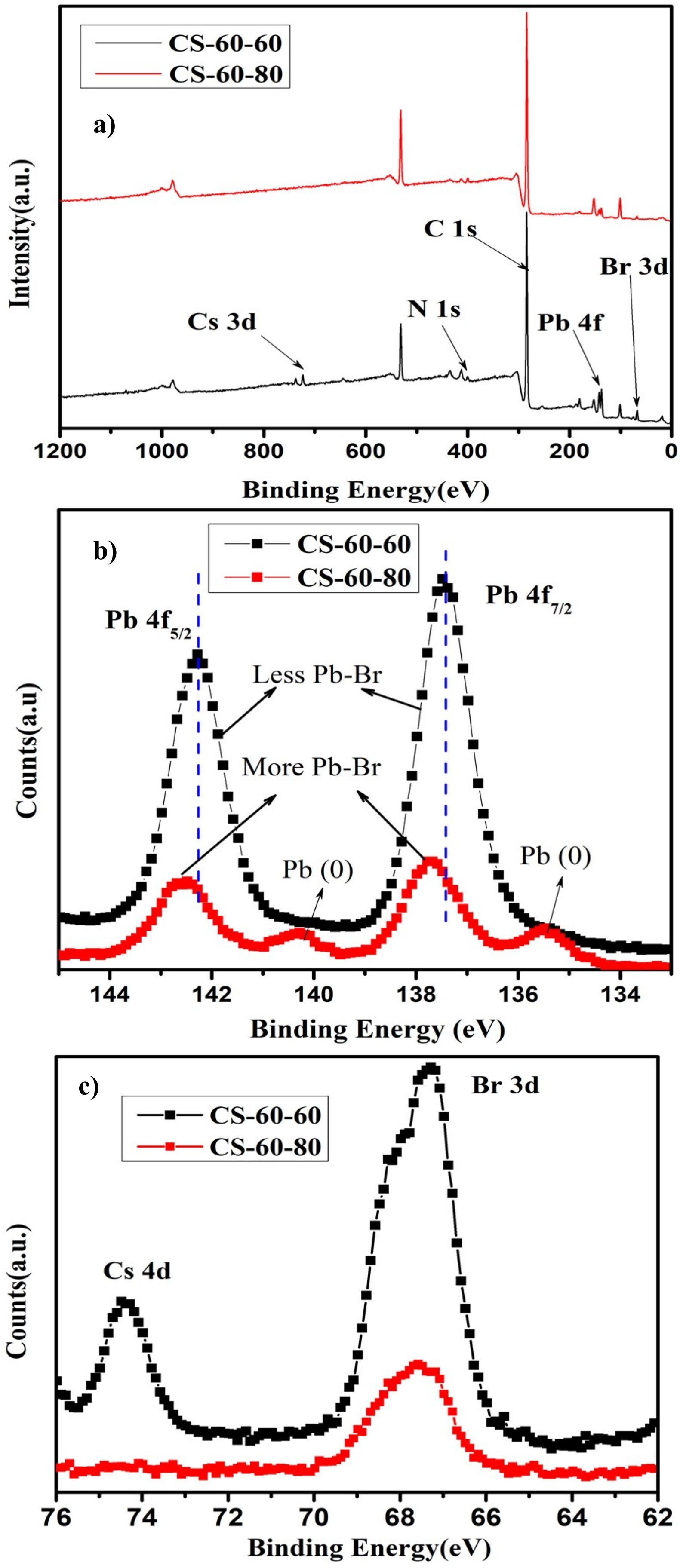
CS-60-60
CS-60-80
a)
Intensity(a.u.)
C 1s
Br 3d
Cs 3d
N 1s
Pb 4f
1200
1000
800
600
400
200
0
Binding Energy(eV)
b)
Pb 4f5/2
Pb 4f7/2
Less Pb-Br
More Pb-Br
Pb (0)
Pb (0)
Counts(a.u
144
142
140
138
136
134
Binding Energy (eV)
c)
Br 3d
Cs 4d
Counts(a.u.)
76
74
72
70
68
66
64
62
Binding Energy(eV)

Figure S22: a) XPS spectra b) High resolution spectra of Pb 4f level and c) High resolution spectra of Br 3d and Cs 4d levels of CS-60-60 and CS-60-80 samples

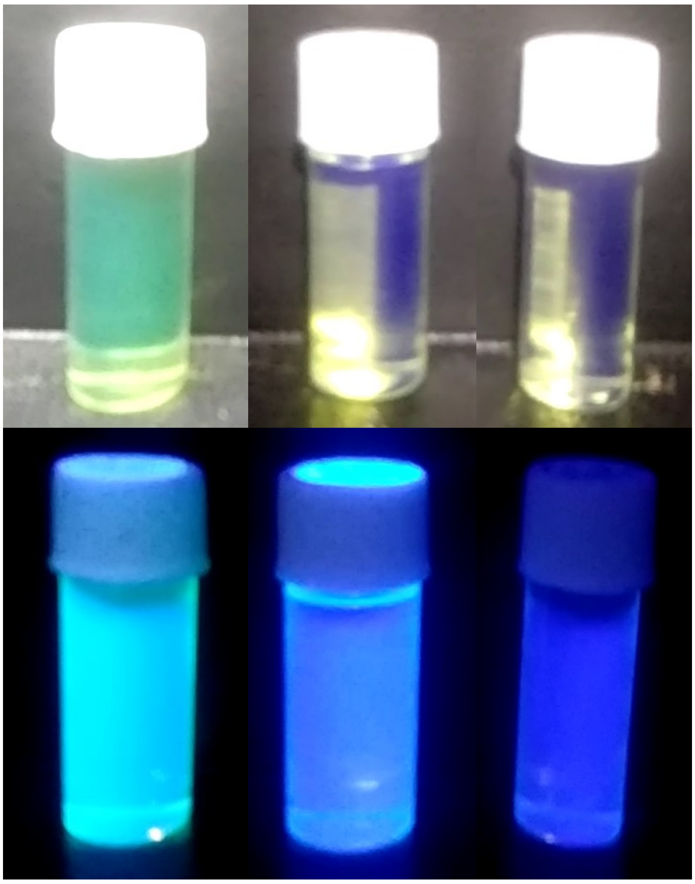

Figure S23: Photographic image of the as-synthesized samples CS-50-50, CS-50-70, CS-50-75 under white light (top) and under UV light (bottom).

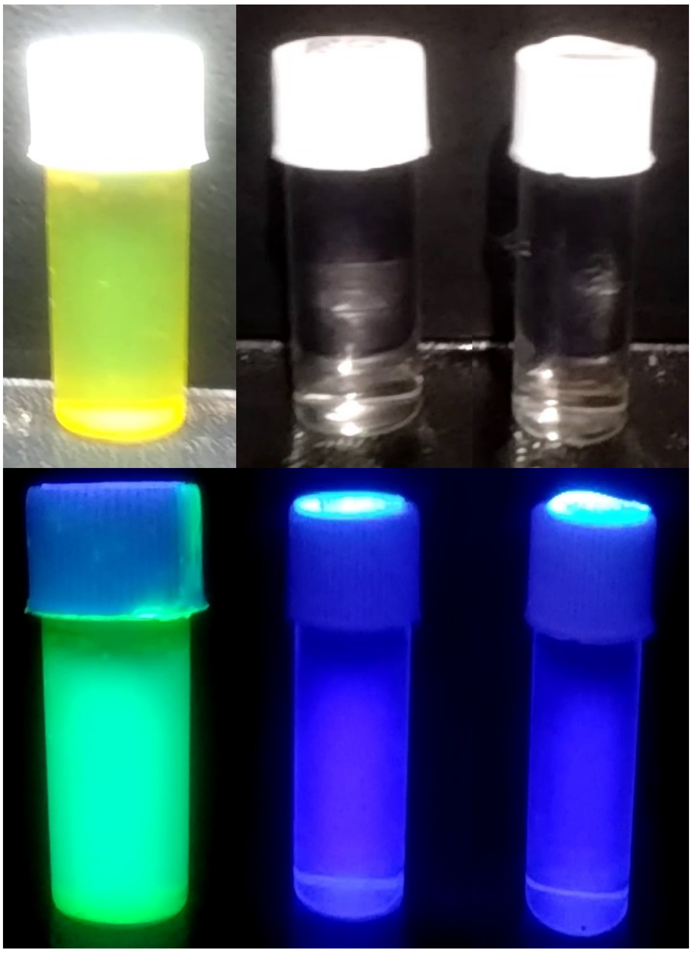

Figure S24: Photographic image of the samples CS-60-60, CS-60-70, CS-60-75, CS-60-80 which are kept in ambient conditions for a month under white light (top) and under UV light (bottom).

Table S4: The short-lived and long-lived PL lifetimes and the distribution coefficients of the samples which are synthesized at 50ºC under different conditions.

Sample	C_1	τ_1	C_2	τ_2	Average
CS-50-50	1.23424	2.01E-08	3.28E+06	3.01E-09	3.01E-09
CS-50-70	0.58295	1.91E-08	965167.8	3.25E-09	3.25E-09
CS-50-75	3.1844	1.33E-08	1.06E+08	2.46E-09	2.46E-09

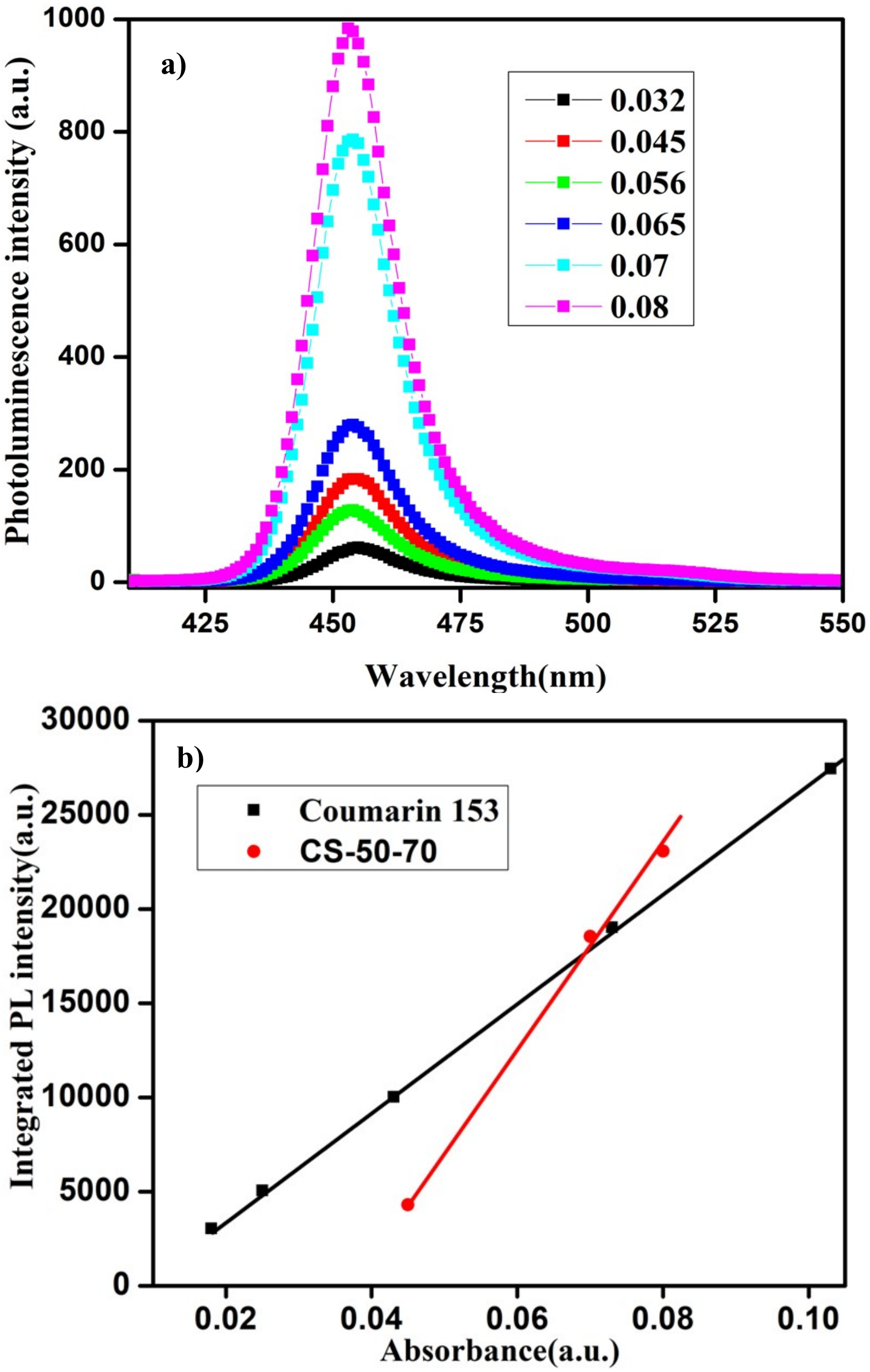

Figure S25: a) PL intensity at an excitation wavelength of 400nm for different values of absorption for the sample CS-50-70 b) Integrated PL intensity vs absorbance for Coumarin 153 and CS-50-70 samples.

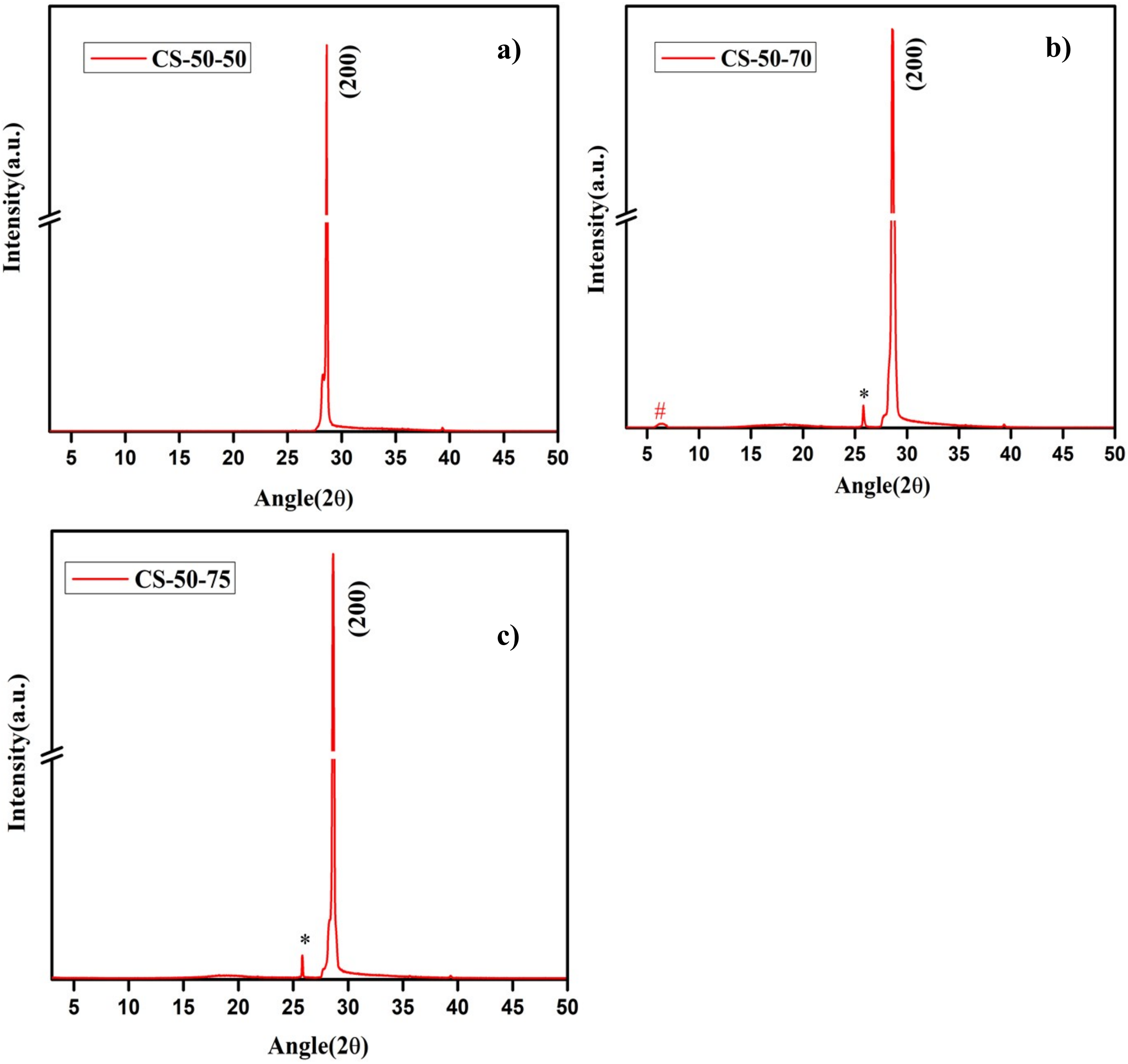

Figure S26: a)-c) XRD spectra of the NPLs which are synthesized at a temperature of 50ºC which are preheated to a different $PbBr_2$ precursor temperature.

In Figure S27a, we compare the FTIR spectra of the sample CS-50-50 and CS-50-70 samples and the FTIR spectra of all the samples are presented in SI Figure S27b. It can be seen from the image for the sample CS-50-50, the peaks were observed at the same wavenumber for the samples synthesized as discussed above. But in the case of the CS-50-75 samples, the intensity of the peaks increases substantially over all wavenumber range. The increase in the intensity of the peaks represents the presence of more functional groups on the

surface of the NPLs. It implies that with the chemical availability of Br the undercoordinated atoms on the decreases, and hence the ligand concentration improves on the surface.

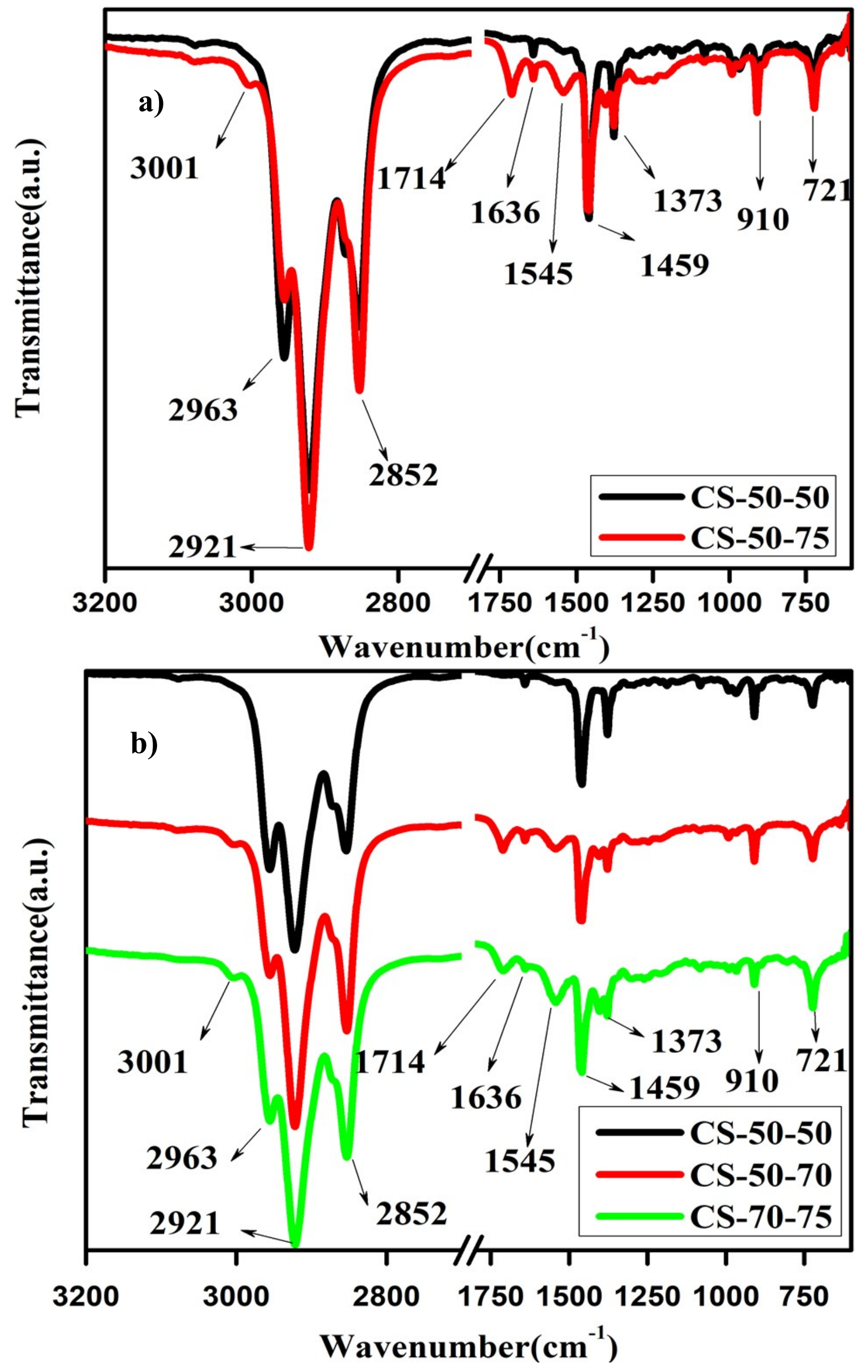

Figure S27: a) Comparison of FTIR spectra of the CS-50-50 and CS-50-70 samples b) FTIR spectra of all the samples which are synthesized at 50ºC.

References

(1) Kim, Y.; Yassitepe, E.; Voznyy, O.; Comin, R.; Walters, G.; Gong, X.; Kanjanaboos, P.; Nogueira, A. F.; Sargent, E. H. *ACS Appl. Mater. Interfaces* **2015**, *7*, 25007.

(2) Kumar, V.; Nagal, V.; Kumar, R.; Srivastava, S.; Gupta, B. K.; Kumar, M.; Hafiz, A. K.; Singh, K. *RSC Adv.* **2020**, *10*, 34651.
